# A Clinical Practice Guideline for Tuberculous Meningitis

**DOI:** 10.1016/S1473-3099(25)00364-0

**Published:** 2025-08-18

**Authors:** Joseph Donovan, Fiona V. Cresswell, Elizabeth W. Tucker, Angharad G. Davis, Ursula K. Rohlwink, Julie Huynh, Regan Solomons, James A. Seddon, Nathan C. Bahr, Arjan van Laarhoven, Suzanne T. Anderson, Sanjay K. Jain, Felicia C. Chow, Sophie Pattison, James E. Scriven, Gabriela Singh, Rob E. Aarnoutse, Jan-Willem C. Alffenaar, Sofiati Dian, Abi Manesh, Robin Basu Roy, Varinder Singh, Ronald van Toorn, Caryn M. Upton, Reinout van Crevel, Kelly E. Dooley, Diana Gibb, David Meya, Robert J. Wilkinson, Ewelina Rogozińska, Usha K. Misra, Anthony Figaji, Guy E. Thwaites

**Affiliations:** 1 Oxford University Clinical Research Unit, Ho Chi Minh City, Vietnam; 2 Department of Clinical Research, London School of Hygiene & Tropical Medicine, London, UK; 3 Global Health and Infection, Brighton and Sussex Medical School, Brighton, UK; 4 MRC/ UVRI-LSHTM Uganda Research Unit, Nakiwogo Road, Entebbe, Uganda; 5 Department of Anesthesiology and Critical Care Medicine, Division of Pediatric Anesthesiology and Critical Care Medicine, Johns Hopkins University School of Medicine, Baltimore, Maryland, USA; 6 Center for Tuberculosis Research, Johns Hopkins University School of Medicine, Baltimore, Maryland, USA; 7 Center for Infection and Inflammation Imaging Research, Johns Hopkins University School of Medicine, Baltimore, Maryland, US; 8 The Francis Crick Institute, Midland Road, London, UK; 9 Wellcome Discovery Research Platforms in Infection, Centre for Infectious Diseases Research in Africa, Institute of Infectious Disease and Molecular Medicine, University of Cape Town, Observatory, Republic of South Africa; 10 Queen Mary and Barts Tuberculosis Centre, Blizard Institute, Queen Mary University London, London, UK; 11 Division of Neurosurgery, Department of Surgery, Neuroscience Institute, University of Cape Town, Republic of South Africa; 12 Centre for Tropical Medicine and Global Health, Nuffield Department of Medicine, Oxford University, Oxford, UK; 13 Department of Paediatrics and Child Health, Faculty of Medicine and Health Sciences, Stellenbosch University, Stellenbosch, Republic of South Africa; 14 Department of Infectious Diseases, Imperial College London, London, UK; 15 Desmond Tutu TB Centre, Department of Paediatrics and Child Health, Stellenbosch University, Cape Town, South Africa; 16 Division of Infectious Diseases and International Medicine, Department of Medicine, University of Minnesota, Minneapolis, Minnesota, USA; 17 Department of Internal Medicine, Radboudumc, Nijmegen, The Netherlands; 18 MRC Clinical Trials Unit, University College London, London, UK; 19 Department of Pediatrics, Johns Hopkins University School of Medicine, Baltimore, Maryland, USA; 20 Departments of Neurology and Medicine (Infectious Diseases), University of California, San Francisco, USA; 21 UCL Library Services, Library, Culture, Collections and Open Science (LCCOS), University College London (UCL), London, UK; 22 Department of Microbes, Infection & Microbiomes, School of Infection, Inflammation and Immunology, College of Medicine and Health, University of Birmingham, Birmingham, UK; 23 Department of Pharmacy, Radboudumc, Nijmegen, The Netherlands; 24 Institute for Infectious Diseases, The University of Sydney, Sydney, New South Wales, Australia; 25 Sydney Pharmacy School, Faculty of Medicine and Health, The University of Sydney, Camperdown, New South Wales, Australia; 26 Westmead Hospital, Department of Pharmacy, Westmead, New South Wales, Australia; 27 Department of Neurology, Faculty of Medicine, Universitas Padjadjaran/Hasan Sadikin Hospital, Bandung, Indonesia; 28 Research Center for Care and Control of Infectious Disease, Faculty of Medicine, Universitas Padjadjaran, Bandung, Indonesia; 29 Department of Infectious Diseases, Christian Medical College, Vellore, India; 30 Blizard Institute, Queen Mary University of London, Newark Street, London, UK; 31 National Center for Excellence in Pediatric Tuberculosis, Department of Pediatrics, Lady Hardinge Medical College and Kalawati Saran Children’s Hospital, Bangla Sahib Marg, New Delhi, India; 32 TASK, Cape Town, Republic of South Africa; 33 Department of Medicine, Vanderbilt University Medical Center, Nashville, Tennessee, USA; 34 Infectious Diseases Institute, College of Health Sciences, Makerere University, Kampala, Uganda; 35 Meta-Analysis Group, MRC Clinical Trials Unit, University College London, London, UK; 36 TS Misra Medical College, Apollo Medics Super Specialty Hospital and Vivekanand Polyclinic and Institute of Medical Sciences, Lucknow, India

## Abstract

Tuberculous meningitis (TBM) is the most severe form of tuberculosis, causing death or disability in around half of those affected. There are no up-to-date international guidelines defining its optimal management. Therefore, the Tuberculous Meningitis International Research Consortium conducted a systematic review of available evidence to address key management questions and develop practice guidance. The consortium includes representatives from India, Indonesia, South Africa, Uganda, Vietnam, Australia, Netherlands, United Kingdom, and United States. Questions were developed using the PICO (Population, Intervention, Comparator, Outcome) format for TBM diagnosis, anti-tuberculosis chemotherapy, adjunctive anti-inflammatory therapy, and neurocritical/neurosurgical care. A GRADE (Grading of Recommendations, Assessment, Development and Evaluations) approach was used to assess certainty (quality) of evidence and determine the direction and strength of recommendations for each PICO question. We provide evidence-based recommendations for the optimal treatment and diagnosis of TBM, alongside expert opinion. We expose substantial knowledge and evidence gaps, thereby highlighting current research priorities.

## Introduction

An estimated 10·8 million people develop tuberculosis (TB) globally each year,(1) of whom 2-5% have tuberculous meningitis (TBM).(2–4) Young children and immunosuppressed individuals, including those living with HIV, are at a particularly high risk of the disease and the associated poor outcomes.(5)

TBM develops following dissemination of *Mycobacterium tuberculosis* from the lungs to the brain. Its clinical course is usually insidious, with typical features of headache, neck stiffness and fever, developing over days to weeks. In untreated cases, neurological deficits develop, consciousness declines and death results.(5) Even with the best available treatment, 20-50% of patients die.(6–10) Early diagnosis and treatment, before the onset of coma, substantially reduces death and disability,(11) yet the best diagnostics and most effective treatments are not well defined. We therefore conducted a systematic review of the available evidence to address a series of predefined, critical clinical questions, to produce an authoritative international practice guideline for the management of TBM.

## Rationale for the guideline

No international clinical practice guideline exists for TBM. Previous national guidelines by the United Kingdom British Infection Society for central nervous system (CNS) TB in adults and children, published in 2009, have not been updated.(12) TB treatment guidelines from the World Health Organization (WHO)(13–15) and jointly from the American Thoracic Society (ATS), Centers for Disease Control and Prevention (CDC), and Infectious Diseases Society of America (IDSA)(16,17) include limited recommendations on CNS TB management. An up-to-date, evidence-based practice guideline is required to help physicians globally provide the best care to adults and children with TBM.

## Guideline scope

We provide guidance for the diagnosis and management of TBM in children and adults, including people living with HIV (PLWH). We provide limited review of other CNS TB complications, including isolated brain tuberculomas, spinal cord TB, and TB brain abscesses. We sought to make recommendations using the best available evidence, providing an assessment of the strength and certainty of our recommendations. However, the recommendations are to guide and do not mandate treatment approaches. Clinicians should continue to exercise their own judgement based upon the individual characteristics of their patients.

## Target audience

The guideline is written for healthcare workers responsible for TBM management anywhere in the world. We recognise that not all diagnostic tests, treatments, and management strategies are available in all settings, and decisions must be individualised by the treating clinician according to available resources.

## Methods

### Guideline inception

The Tuberculous Meningitis International Research Consortium identified the need for a TBM practice guideline in October 2020. A writing group was convened from within the multidisciplinary and global Consortium to define its scope, target audience, and methods.

Four working groups addressed key questions concerning diagnosis, anti-TB chemotherapy, adjunctive therapy, and neurocritical/neurosurgical care. Individuals were assigned to working groups (4-6 members/group) based on expertise and experience, ensuring adult and paediatric expertise within each group. A Guideline Steering Group provided oversight. Working groups were supported by a librarian and methodologist.

### Questions

Key questions were developed using the PICO (Population, Intervention, Comparator, Outcome) format. Final questions, with recommendations, are shown in boxes 1-4.

### Search strategy and selection criteria

Literature searches were conducted for each PICO question using keywords and controlled vocabulary. Ovid Medline, Embase, Cochrane CENTRAL, Global Health and Global Index Medicus were searched from inception until 24th July 2023, followed by a final screening for new literature on 11th March 2025 (appendix p34). Search strategies for Medline are provided in appendix pp26-32. A total of 35,143 records were retrieved, with 7380 records screened for relevance after duplicate removal. Non-English language articles and conference abstracts were excluded.

### Study selection

For diagnostic questions, we only included test accuracy studies. For anti-TB chemotherapy, only data from phase II/III randomised trials using standard WHO-recommended therapy as the comparator were considered for adults. Pharmacokinetic (PK) studies not reporting a mortality endpoint were excluded. An up-to-date systematic review and meta-analysis of anti-TB chemotherapy in children directly informed recommendations;(18) a literature review was not repeated. For optimal antiretroviral therapy (ART) timing, only randomised controlled trials (RCTs) were included. For neurocritical/neurosurgical care, a ‘standard WHO therapy’ comparator included only studies after 1995, when streptomycin was phased out as a first-line drug (ensuring recommendations were relevant to current treatment).

For all questions, abstracts were independently assessed by two reviewers from working groups and relevant abstracts were shortlisted for full text review. When the reviewers did not agree on abstract inclusion, consensus was reached by discussion. Full texts of included studies were retrieved and independently assessed for eligibility by two reviewers. Data extracted from eligible studies was tabulated and quality assessed. Full text data extraction was performed by one group member, with data from a random sample of 10% of studies cross-checked by another group member.

### Certainty of evidence

A GRADE (Grading of Recommendations, Assessment, Development and Evaluations)(19) approach was used to assess certainty (quality) of evidence and determine the direction and strength of recommendations. Data for PICO questions were summarised and presented in summary of findings tables (appendix pp3-18 & 19-25). The following domains were assessed: risk of bias (using a standard approach to applying signalling questions), indirectness, inconsistency, imprecision, and other considerations (including publication bias). Risk of bias assessment was performed using appropriate tools (e.g., Quality Assessment of Diagnostic Accuracy Studies 2 [QUADAS-2] for diagnostic studies, Revised Cochrane Risk-of-Bias tool for Randomized Controlled Trials [RoB2] for RCTs).(20,21) Certainty assessment was performed by one individual from the respective working group, with certainty downgrading/upgrading performed in line with the GRADE approach.(22)

For each PICO question, ‘certainty of evidence’ (high, moderate, weak, very weak) and ‘strength of recommendation’ (strong or weak, for or against) were stated.(19) Justifications for recommendation strengths are given in accompanying narrative. Draft recommendations were developed and approved in guideline group meetings. Wider consultation with global TBM experts (~120 people) occurred during Tuberculous Meningitis International Research Consortium Meetings (Oxford, UK, September 2023, and Bali, Indonesia, November 2024). An evaluation of patient values and preferences was not performed.

### Limitations

Only English language articles were included; studies from high-burden countries not published in English were not included. Quality assessment was performed by an individual researcher from each working group (rather than two independent researchers).

### Good practice points

**Figure 1:**
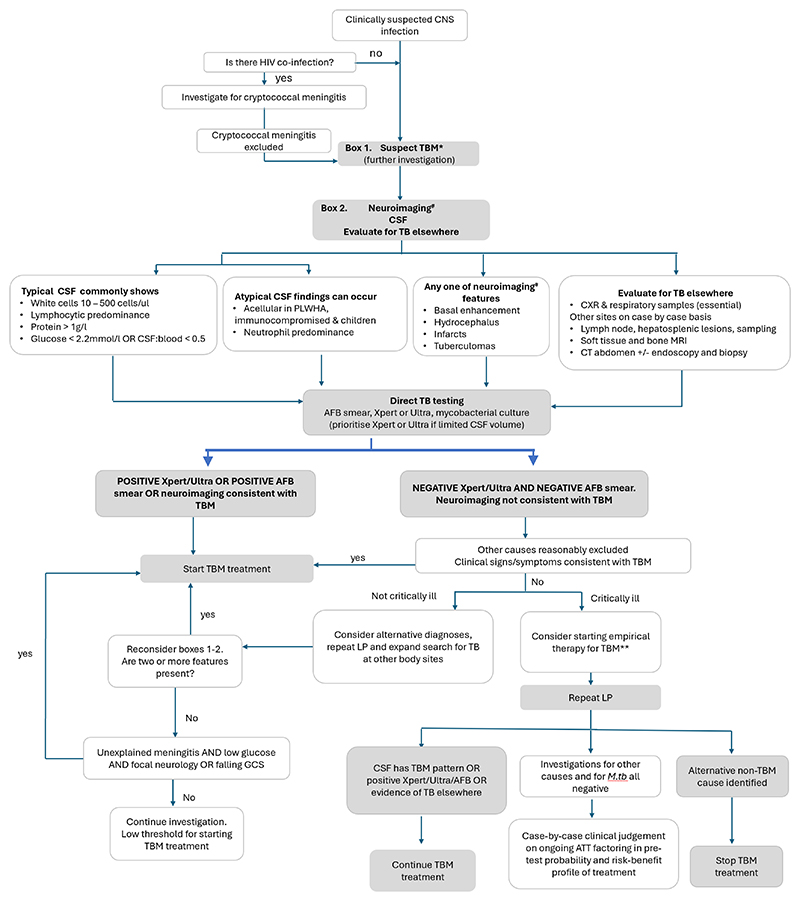
Diagnostic approach for suspected TBM in children and adults **Boxes shaded in grey represent evidence-based recommendations. Boxes without shading represent consensus recommendations drawn from collective expert opinion and expertisen.** In PLWHA, cryptococcal meningitis can present similarly to TBM and should be excluded in the first instance as CrAg testing is highly sensitive.(129) Large volumes of CSF are recommended. Diagnostic test(s) performed will depend on local availability, however multiple TB testing where available should be performed. Where rapid diagnostic testing or neuroimaging is consistent, ATT for TBM should be commenced. Whilst mycobacterial culture does not return rapid results, this remains an important diagnostic test to perform. Where rapid diagnostic testing is negative and neuroimaging is not suggestive of TBM, a decision to start treatment should be made on degree of clinical suspicion, repeated evaluations, neurological deterioration and active exclusion of other possible causes. **Top: Diagnosis**. Box 1. Suspect TBM* where risk factors, symptoms, and signs, are suggestive. *Risk factors other than HIV include immunosuppression, malnutrition, travel or residence in TB endemic region, young age, contact with infectious TB in the last 1-2 years. Compatible symptoms and signs include >5 days of fever with any of: headache, vomiting, neck stiffness, poor appetite or poor weight gain (young children), cough, cranial nerve palsy. Box 2. Mass lesions and raised intracranial pressure may develop as part of CNS tuberculosis (tuberculoma or tuberculous abscess) or from an alternative diagnosis (e.g., brain tumour or bacterial abscess); as such in patients being evaluated for TBM there may be contraindications to lumbar puncture due to the risk of cerebral herniation. Neuroimaging^#^ should be performed before lumbar puncture (to exclude the risk of herniation) if this is possible, and lumbar puncture only performed when it is safe to do so. Obtaining neuroimaging before lumbar puncture may delay treatment initiation,(130) therefore clinical discretion should be used on a case-by-case basis. Modality of neuroimaging^#^ depends on availability. CT is often accessible and, using contrast, can detect hydrocephalus, basal exudates, large infarcts and tuberculomas. MRI is more sensitive at detecting small and evolving infarcts particularly in the brainstem. **Bottom: Treatment initiation**. **Differentiating TBM from other meningitides in high incidence TB settings can be challenging. Strongly consider starting empirical therapy in conjunction with treatment for alternative causes in the critically unwell. Repeating CSF analysis with TB testing may provide valuable guidance when deciding between TBM or other CNS infection. In the absence of a perfect test to diagnose TBM, clinical judgement on whether to initiate or continue anti-TB treatment should consider all aspects of the case including epidemiological, clinical, laboratory, and imaging features where this is available. Specialist input should be sought. AFB=acid-fast bacilli. ATT=anti-tuberculosis chemotherapy. CNS=central nervous system. CSF=cerebrospinal fluid, HIV=human immunodeficiency virus. LP=lumbar puncture. M.tb=*Mycobacterium tuberculosis*. OP=opening pressure. PLWHA=people living with HIV/AIDS. TBM=tuberculous meningitis.

**Figure 2:**
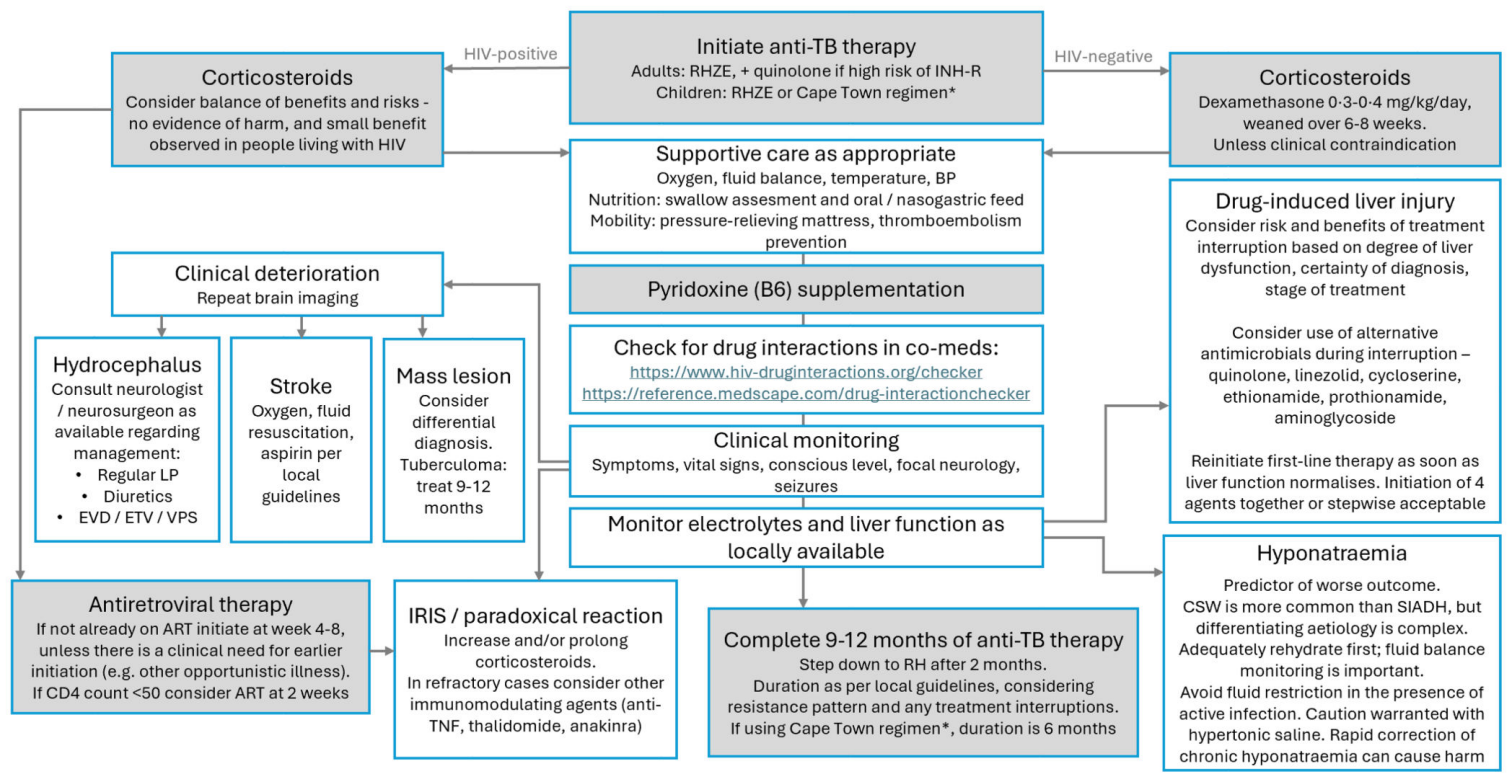
Summary of the treatment and follow-up of adults and children, with or without HIV, with TBM Boxes shaded in grey represent evidence-based recommendations. Boxes without shading represent consensus recommendations drawn from collective expert opinion and expertise. *The Cape Town regimen is also WHO-recommended for the treatment of drug-susceptible TBM in children and is a 6-month duration with elevated dosages of isoniazid 15-20mg/kg/day, rifampicin 22·5-30mg/kg/day and pyrazinamide 35-45mg/kg/day, and the substitution of ethambutol with ethionamide 17·5-22·5mg/kg/day. ART=antiretroviral therapy. BP=blood pressure. CSW=cerebral salt-wasting syndrome. EVD=external ventricular drain. ETV=endoscopic third ventriculostomy. INH-R=isoniazid resistance. IRIS=immune reconstitution inflammatory syndrome. LP=lumbar puncture. SIADH=syndrome of inappropriate antidiuretic hormone secretion. TNF=tumour necrosis factor. VPS=ventriculoperitoneal shunt.

Recognising the need to provide practical guidance, even when evidence is limited or absent, we also provide expert-opinion-based ‘good practice points’ and figures (figures 1&2) summarising suggested diagnostic and therapeutic approaches.

## Recommendations

### Diagnosis of TBM

Recommendations are shown in box 1, with evidence synthesis in appendix pp3-13.

A uniform case definition was created in 2010 as a research tool for TBM classification, defining definite, probable, possible and not TBM.(23) Developed as a standardised approach to classifying TBM diagnosis in research, it is now used as a standard against which diagnostic tests are assessed.

#### Rationale and context for recommendations

**1. How accurate are cerebrospinal fluid (CSF) microscopy and biochemistry to diagnose TBM?**

We found no studies directly addressing this question. The TBM lumbar CSF profile typically includes a moderate lymphocytic pleocytosis, elevated protein, low glucose (<50% plasma concentration) and moderately elevated lactate (5-10mmol/l).(24) However, none of these parameters is specific enough individually, or in combination, for definitive diagnosis. However, CSF analysis is an essential part of the TBM diagnostic workup, providing supporting evidence for TBM diagnosis(28) and an opportunity to detect *M. tuberculosis* or identify other causes of meningoencephalitis.

**2. How accurate are microbiological and molecular tests to diagnose TBM?**

The challenge in detecting *M. tuberculosis* within CSF is the very low bacterial numbers, limiting the sensitivity of all currently available tests. Technician skill, large CSF volumes and optimal processing improve diagnostic yields.(25–27)

In most settings, CSF Ziehl-Neelsen (ZN) smear is insensitive (<30%), and provides little advantage over Xpert MTB/RIF (Xpert) or Xpert MTB/RIF Ultra (Ultra) (GeneXpert, Cepheid Sunnyvale, CA) (appendix pp3-4).(27) Xpert and Ultra are PCR-based tests that provide rapid results and detect mutations associated with rifampicin resistance. Ultra’s lower limit of detection enhances its sensitivity making it the test of choice, when available.(28) Both Xpert and Ultra demonstrated high specificity (appendix pp5-8), although neither test can rule out TBM. These tests should, when possible, be combined with mycobacterial culture, enabling subsequent extended drug susceptibility tests (appendix p9). The accuracy of Xpert and Ultra to detect rifampicin resistance reduces when bacterial numbers are very low.(29)

Alere-Lipoarabinomannan (LAM) (Abbott Determine TB-LAM antigen, Lake Bluff, USA) identifies *M. tuberculosis* cell wall components by lateral flow. There is insufficient evidence to recommend for or against using Alere-LAM on CSF (appendix pp10-11).

**3. How accurate is adenosine deaminase (ADA) to diagnose TBM?**

ADA accuracy assessment is limited by variable assays with uncertain positive test cut-offs, and lack of gold standard comparators (appendix pp12-13). Elevated CSF ADA concentrations are not specific for TBM. Whilst evidence certainty was very low, ADA measurement is relatively inexpensive, and elevated concentrations may prompt utilisation of better tests (e.g. Ultra). Measurement of CSF ADA should not replace Xpert, Ultra, or culture.

**4. How accurate is neuroimaging to diagnose TBM?**

We found no studies directly assessing this question. However, brain imaging, with CT or MRI, enables assessment of the incidence and evolution of the common TBM complications (hydrocephalus, tuberculomas, infarctions) before and after the start of TBM treatment.(30) For these purposes, baseline brain imaging is recommended.

#### Good practice points

##### CSF volume

We recommend sampling ≥6mLs CSF for dedicated *M. tuberculosis* testing.(25) Larger CSF volumes are a strong predictor of positive ZN stain, *M. tuberculosis* culture, and Xpert.(26,31) CSF should be centrifuged at 3000g for 20 minutes, with the cell pellet subject to mycobacterial tests.(25,31,32)

##### Children and PLWH

Our recommendations apply to all age groups and PLWH. Only one study evaluated Ultra in children, reporting 50% sensitivity,(33) lower than adult studies (~65%), probably reflecting lower CSF volumes from children. Sensitivities of ZN stain, mycobacterial culture, Xpert and Ultra are higher in PLWH, probably due to higher bacillary loads.(34–36)

##### Diagnostic approach and empirical therapy

No single negative test can rule out TBM. Combining CSF ZN smear, Xpert/Ultra, mycobacterial culture, can increase diagnostic yields.(34,36) Repeated testing of CSF may increase diagnostic yields. Consistent neuroimaging features, e.g. hydrocephalus, basal exudates, infarcts or tuberculomas, increases the probability of TBM,(30,37,38) as does *M. tuberculosis* identification outside the CNS. Testing of sputum for *M. tuberculosis* is recommended given pulmonary TB is present in ~50% of cases of TBM.(9,10) Nevertheless, given the limited sensitivity of available tests and the fatal consequences of delayed treatment, many patients (30-50%) must start treatment empirically,(6,9,10,39) based on clinical suspicion alone. A diagnostic approach to TBM, based on evidence and expert opinion, is presented in figure 1.

### Anti-TB chemotherapy

Recommendations are shown in box 2, with evidence synthesis in appendix 14-18.

Anti-TB drug regimens for TBM treatment are based on those developed for pulmonary TB and do not account for the need to achieve therapeutic concentrations within the CNS.(40) TBM caused by bacteria resistant to critical first-line drugs (rifampicin and isoniazid) is an increasing therapeutic challenge, with few data describing the CNS activity and effectiveness of drugs recently approved and highly effective for multi-drug resistant pulmonary TB treatment.(41–44) Recent animal and clinical studies utilising PET imaging with radiolabelled antibiotics has provided insights on CSF and brain distribution, and activity of these drugs.(45–48)

#### Rationale and context for recommendations

**1. Does increasing the rifampicin dosing reduce mortality in adults with TBM vs. standard 10mg/kg/day dosing?**

Standard 10mg/kg/day rifampicin results in very low CSF concentrations/exposures.(9,49) Rifampicin was undetectable in CSF in two-thirds of patients with TBM in two studies using the standard dose.(7,50) Higher rifampicin doses/exposures increase bacterial killing in pulmonary TB,(51) and 35mg/kg/day has been safely used in adults and children with pulmonary TB and TBM.(52–55)

Four phase II and one phase III trial have investigated higher rifampicin doses for TBM (appendix pp14-15). Across these five studies, three studies did not have mortality as their primary outcomes or utilised high-dose rifampicin with other interventions (linezolid and aspirin).(42,50,56) The duration (2-8 weeks) and doses (15-35mg/kg/day) of rifampicin varied, but higher rifampicin doses were not associated with reduced TBM mortality (odds ratio 0·91, 95% confidence interval [CI] 0·56-1·46). However, these data are dominated by a large (817 adults) phase III trial investigating 15mg/kg/day rifampicin, which may not have resulted in sufficiently high CSF exposures.(9,49) We therefore examined the benefit of rifampicin >20mg/kg/day, including lower dose intravenous administration that achieved equivalent exposures. Data were limited and a mortality benefit was not observed, although >20mg/kg/day was safe.

Two active phase III trials are investigating 35mg/kg/day rifampicin in adults with TBM. Later in 2025, the HARVEST trial (ISRCTN15668391) will report results, and the INTENSE-TBM trial (NCT04145258) will report results in 2026. The results of these trials are likely to provide definitive data to address this PICO question.

**2. Does an adjunctive fluoroquinolone or linezolid reduce mortality in adults from TBM?**

Five RCTs have evaluated the addition of fluoroquinolones to standard rifampicin-based regimens for TBM (appendix p16). Three studied levofloxacin, one moxifloxacin, and one levofloxacin, ciprofloxacin and gatifloxacin. Taken together, the addition of a fluoroquinolone to the regimen was not associated with significantly reduced mortality (odds ratio 0·86 95% CI 0·51-1·45). However, post-hoc analysis of the 2016 Vietnam trial(9) found rifampicin 15mg/kg/day with levofloxacin (as the fifth drug) reduced mortality in adults with TBM caused by isoniazid resistant bacteria (hazard ratio 0·34, 95% CI 0·15-0·76, P=0·01).(57)

The WHO endorsed linezolid for multi-drug resistant (MDR) pulmonary TB treatment in 2019.(14) Data investigating its use in TBM treatment are limited to three small phase II trials (appendix p17),(42,58,59) which did not establish its benefit in presumed drug-sensitive TBM. Linezolid may, however, have a role in treating MDR-TBM, given its favourable CNS PK and bactericidal activity.(60)

**3. Does higher dosing, or alternative administration routes, of other TB drugs reduce mortality in adults from TBM?**

Only one study has addressed this question: a phase II trial of higher-dose intravenous isoniazid (500mg/day) and ethambutol (2g/day) with rifampicin and pyrazinamide in 54 adults (appendix p18).(61) Pharmacokinetic analysis of the 2016 Vietnam trial found a strong association between high CSF isoniazid concentrations, slow acetylator status, and reduced case-fatality.(49) A 6-month regimen with high doses of rifampicin, isoniazid, pyrazinamide (RHZ) and ethionamide has produced excellent outcomes in South African children (see PICO question 5), but has not been studied in adults.

Intrathecal administration, usually of aminoglycosides, were used in the early years of anti-TB chemotherapy,(62) but became uncommon once RHZ became available. It has some recent proponents,(63,64) although there are no comparative trials and data are insufficient to make a recommendation.

**4. Is treatment duration less than 12 months effective in TBM in adults?**

No trials comparing <12 months with ≥12 months anti-TB chemotherapy were identified. In 2016, a meta-analysis of 19 observational studies concluded that in all cohorts most deaths occurred in the first six months; and relapse was uncommon in all participants irrespective of the regimen. No inferences regarding optimal treatment duration could be made.(65) The WHO currently recommend treating adult TBM with 12-months of anti-TB drugs; there is no evidence supporting a different recommendation. Additionally, there is no substantive evidence to support longer durations of anti-TB chemotherapy for TBM (than for pulmonary TB) in terms of better outcomes.

**5. What is the optimal treatment for childhood TBM?**

Due to the non-linear effect of weight on clearance, young children (particularly <2 years) achieve lower drug exposures when dosed at the same mg/kg dosage as older children, adolescents and adults.(66) Rifampicin up to 35mg/kg/day is safe in children, and in one study, doses of up to 65-70mg/kg rifampicin were needed to reach the target exposure in children.(53) Additionally, a small phase II RCT of children with TBM reported better neurocognitive outcomes in those receiving regimens containing high-dose (30mg/kg/day) rifampicin.(67) For more than 30 years, children with TBM in South Africa have been treated with 6 months of higher doses of isoniazid, rifampicin, pyrazinamide and ethionamide, with excellent outcomes.(68) A recent systematic review informed the 2022 WHO child and adolescent TB guidelines;(18) no clinical trials were identified but 7 observational studies provided evidence, graded as low quality. In children and adolescents ≤19 years with drug-susceptible TBM, WHO recently recommended that a 6-month regimen (isoniazid 15-20mg/kg/day, rifampicin 22·5-30mg/kg/day and pyrazinamide 35-45mg/kg/day, and the substitution of ethambutol with ethionamide 17·5-22·5mg/kg/day) can be used instead of the 12-month standard regimen.(69)

Results of the SURE trial (ISRCTN40829906),(70) a RCT comparing a 6-month intensive regimen to the 12-month standard for childhood TBM, will be available by the end of 2025.

#### Good practice points

##### Drug-resistant TBM

Mortality from TBM caused by bacteria resistant to rifampicin and isoniazid (MDR TBM) exceeds 70%.(57,71) Poor outcomes are driven by delayed detection of resistance and initiation of second-line anti-TB treatment, compounded by the uncertain effectiveness of second-line drugs in TBM. There are no RCTs informing guidance.

Early rifampicin resistance detection is critical to outcomes. Therefore, Xpert or Ultra testing of CSF and other specimens, if extra-neural TB is suspected, are strongly encouraged in all patients with TBM. Clinical deterioration after the start of anti-TB treatment is an unreliable indicator of MDR TBM as it is more commonly caused by hydrocephalus, infarcts, or other inflammatory complications (e.g. tuberculomas).

Without trials, the selection of second-line drugs is based upon their predicted activity within the CNS (appendix p19). CSF pharmacokinetic data assist that selection, although CSF concentrations of some drugs do not correlate well with brain concentrations. For example, rifampicin, delamanid, pretomanid and bedaquiline achieve much higher concentrations in brain than CSF.(45–47,72) Employing therapeutic drug monitoring to address low exposure in plasma/serum may help to optimise CNS concentration.(73)

The BPaL regimen is highly effective for MDR pulmonary TB,(74) but it has not been evaluated in patients with MDR TBM. In animal models of TBM, the BPaL regimen is inferior to the standard TB regimen,(45) with no additive effective of bedaquiline.(48) Excellent activity was however noted in animal studies with PaZ-based regimens such as PaLZ.(48) Whether bedaquiline achieves sufficient CNS exposure to be effective is uncertain. However, ≥50% of patients with TBM have concurrent pulmonary TB,(9) for which bedaquiline will be highly effective.

##### Adverse drug effects

Pyrazinamide, isoniazid and rifampicin can all cause drug-induced liver injury (DILI), the commonest reason for treatment interruption and drug substitution. However, unlike in pulmonary TB, stopping anti-TB drugs is an independent risk factor for death from TBM.(75–77) Therefore, clinicians should weigh carefully the risks of discontinuation of anti-TB therapy with the severity of DILI.

Little evidence exists to guide clinicians, although the ACT HIV(10,77) and LAST ACT(76) (NCT03100786) TBM trials randomised participants who developed DILI to three strategies: replace RHZ with a fluoroquinolone and an aminoglycoside; withdraw pyrazinamide and monitor transaminases; or continue all drugs unless transaminases rise >10x normal. Results are anticipated by the end of 2025.

Reintroduction of first-line drugs can be considered once liver function normalises, either stepwise or all at once, at a full dose or an escalating dose. There is insufficient evidence to support one approach over another, or the order of the drugs to be reintroduced.(78)

Drug-drug interactions are an essential consideration in the treatment of TB. Rifampicin induces the hepatic metabolism of a wide variety of drugs, including ART. The most up-to-date information on drug interactions can be found at https://www.hiv-druginteractions.org/checker.

### Adjunctive therapy

Recommendations are shown in box 3, with evidence synthesis in appendix pp21-24.

Outcomes from TBM are strongly associated with dysregulated inflammatory responses.(79) However, these responses vary substantially between individuals. For example, PLWH and TBM have higher concentrations of inflammatory markers but lower numbers of leucocytes, compared to TBM patients without HIV.(39,80)

Adjunctive corticosteroids have been given to control TBM-associated inflammation ever since anti-TB drugs became available to treat TBM.(81) The challenge, however, has been recognising the heterogeneity in inflammatory response and identifying those who benefit most from corticosteroids or, more recently, better targeted adjunctive therapies.

#### Rationale and context for recommendations

**1. Should corticosteroids be used as an adjunctive therapy in patients with TBM?**

Corticosteroids are recommended by WHO and ATS/CDC/IDSA for everyone with TBM, regardless of severity.(16,82) The results of two large (n=1065) and seven smaller (n=585) RCTs, support these and our recommendations (appendix pp21-22). Corticosteroids reduced case-fatality, especially in children and adults without HIV.(83) There is no signal for a change in disability amongst survivors in these groups.

In PLWH and TBM, the benefits of corticosteroids are uncertain. One large RCT, published 3 months after the updated literature search (23/7/2023), was included given its relevance.(10) 520 adults with HIV-associated TBM were enrolled; dexamethasone was associated with a non-significant survival benefit (hazard ratio 0·85; 95% CI 0·66-1·10). Disability and the incidence of immune reconstitution inflammatory syndrome (IRIS) were not reduced by dexamethasone. Despite most participants being profoundly immune-suppressed (52% CD4 <50 cells/mm^3^), dexamethasone did not increase adverse events.

In the absence of an effective alternative adjunctive therapy for HIV-associated TBM, and the safety and potential effectiveness of corticosteroids, we recommend their use on a case-by-case basis in PLWH.

**2. What is the optimal timing of antiretroviral therapy in CNS TB?**

Clinical trials in PLWH with pulmonary TB have demonstrated clear mortality benefit for patients with CD4 counts <50/mm^3^ who start ART within 2 weeks of starting anti-TB treatment, albeit with increased risk of IRIS.(84–86) One RCT has been conducted in PLWH with TBM (median CD4 count 41cells/mm^3^) comparing ART initiation within 7 days of anti-TB treatment or at 2 months. No difference in 9-month survival between the arms was found (appendix p23).(87) This finding was similar across all CD4 counts. More grade 4 laboratory events were observed in the immediate ART group, but no increase in neurological events.

These limited data inform our weak recommendation to defer ART for 4-8 weeks after starting TB treatment. This is in agreement with WHO and other guidelines.(88–91) A range has been given based on expert opinion and clinicians should decide to start ART based on individual patient factors, considering CD4 count (if available), other opportunistic infections, neuroimaging and TBM-IRIS risk factors (CSF ZN/culture positivity, CSF neutrophil pleocytosis).(92)

**3. What other adjunctive therapies can be considered for the management of TBM?**

Several small phase II studies in adults and children suggest aspirin, added to corticosteroids, may reduce the incidence of brain infarcts and death (appendix p24). However, the trials are too small and heterogenous to be definitive and a recommendation to use aspirin routinely cannot be given. The SURE (ISRCTN40829906) and INTENSE-TBM (NCT04145258) trials, investigating adjunctive aspirin in children and adults respectively, will provide high-quality data from the end of 2025.

Observational studies in South African children have suggested adjunctive thalidomide (2-5mg/kg/day) was safe and effective in treating TB mass lesions and optochiasmatic arachnoiditis.(93,94) A trial of higher dose thalidomide (24mg/kg/day) was terminated early due to adverse effects and mortality in the thalidomide arm.(95) No additional trials have been reported. Teratogenicity and other adverse events have limited thalidomide’s use as an adjunctive agent.

Case series have suggested biological agents targeting TNF-α (e.g. infliximab, adalimumab) can help treat tuberculomas and optochiasmatic arachnoiditis.(96,97) A retrospective cohort study in India reported adjunctive infliximab (10mg/kg 1-3 doses, 4 weeks apart) was safe and effective in treating severe inflammatory complications of TBM.(98) The active TIMPANI trial (NCT05590455) is investigating adjunctive adalimumab in adults with TBM and HIV.

### Good practice points

#### Paradoxical reactions and IRIS

Adjunctive anti-inflammatory therapies (e.g. corticosteroids) are usually given with anti-TB drugs at the start of treatment. However, in ~20% of patients with TBM (>30% PLWH), inflammatory intracerebral complications occur. These typically arise after 20-60 days of treatment, but can occur many months later. Often called ‘paradoxical reactions’, they occur despite effective anti-TB treatment. In the context of PLWH starting ART, they can meet the criteria for IRIS,(99) although the clinical and imaging characteristic are similar, regardless of HIV status.

The management of these inflammatory complications has not been subject to trials. Expert opinion recommends using high-dose corticosteroids initially (e.g. dexamethasone 0·4mg/kg/day), tapering slowly according to symptom resolution. If corticosteroids do not control symptoms, then small case-series/case reports have described the use of anti-TNF biologics (e.g. infliximab),(98) thalidomide,(100) or anakinra.(101,102)

Adjuvant interferon-gamma treatment has been described in refractory CNS tuberculosis,(103) and cyclophosphamide treatment described in CNS vasculitis.(104,105) Data are too limited to make recommendations concerning their use (appendix p35).

### Neurocritical and neurosurgical care

Recommendations are shown in box 4, with evidence synthesis in appendix p25.

TBM causes critical illness with unique neurocritical and neurosurgical considerations. Raised intracranial pressure (ICP) can fatally compress brain tissue and cause ischaemia. Cerebral infarction is common (>65%) and predictive of poor outcomes.(106) Key management concerns relate to controlling raised ICP, whether from oedema, hydrocephalus, or mass lesions (tuberculomas/TB abscess), and ameliorating cerebral ischaemia from raised ICP and vasculitis.(107)

#### Rationale and context for recommendations

**1. Should active management (medical and/or surgical) or standard of care be used in individuals with TBM and hydrocephalus/raised ICP?**

Hydrocephalus occurs in 50-90% of patients. Management varies from monitoring without intervention, to medical (regular lumbar punctures and/or diuretics) and neurosurgical (lumbar or external ventricular drains [EVD], endoscopic third ventriculostomy [ETV] or ventriculoperitoneal shunts [VPS]) intervention.(106,108–111) No studies have directly compared these approaches. Lack of standardised definitions and management approaches preclude evidence-based recommendations.

**2. Should a VPS or ETV be used for the surgical management of hydrocephalus in patients with TBM?**

Two single-centre RCTs have compared VPS with ETV.(112,113) Whilst both studies demonstrated the benefit of surgical intervention, mortality and success rates were similar between the interventions (appendix p25). These procedures should be considered on a case-by-case basis; recommending one procedure over the other cannot be made based on the available data.

**3. Should surgical management of tuberculomas with or without TBM occur at time of diagnosis or after medical treatment failure? 4. Should surgical management of TB abscesses with or without TBM occur at time of diagnosis or after medical treatment failure?**

No studies directly address these two PICO questions. Surgery can be necessary, but no studies have compared the timing of surgery for tuberculomas or TB abscesses. There was heterogeneity in diagnosis, surgical techniques (biopsy vs. debulking vs. full resection), lesion location, duration of follow-up, and assessment of resolution or treatment failure. Evidence-based recommendations cannot be made. Consortium members have reviewed the management of intracranial TB mass lesions elsewhere.(114)

**5. Should the management of hyponatremia in patients with TBM be based on aetiology?**

Hyponatremia can cause cerebral oedema, raised ICP and infarction.(107,115–117) Studies suggest it is more commonly caused by cerebral salt wasting (CSW) than syndrome of inappropriate antidiuretic hormone secretion (SIADH), although their discrimination is difficult and diagnostic criteria vary.(117,118) Whether outcomes are improved by management tailored to cause is uncertain. There are insufficient data to make recommendations concerning optimal management of TBM-associated hyponatraemia.

**6. Should all patients with TBM be assessed for clinical and sub-clinical seizures?**

Seizures can occur due to raised ICP, tuberculomas and ischaemia, and can increase cerebral oxygen consumption increasing the risk of a metabolic crisis and infarction.(119,120) The pooled incidence of EEG-confirmed seizures was 25% from five descriptive studies; they are more common in children than adults.(121–125) Seizures are associated with increased mortality and morbidity.(122,126) We found no studies directly addressing the PICO question. Therefore, we were unable to provide recommendations.

#### Good practice points

##### Supportive care and checklists

A comprehensive assessment proforma and an accompanying ‘priorities’ checklist for patients with TBM were proposed in 2019 by Consortium members.(127) The proforma outlines what should be asked, checked, or tested at initial evaluation and daily inpatient review to assist supportive clinical care for patients. The checklist offers a useful and easy reminder of important issues to review during a time-critical period of acute patient deterioration. A global survey demonstrated many centres (>90%) have the resources to apply these approaches.(127,128)

Figure 2. provides an evidence-based and expert opinion overview of TBM treatment.

## Supplementary Material

appendix

## Box 1 Diagnostic recommendations by PICO question

**1. How accurate are CSF microscopy and biochemistry to diagnose TBM?**

**P**opulation: All individuals in hospital being evaluated for TBM

**I**ntervention: CSF cell microscopy, biochemistry, lactate

**C**omparators: a) Definite or probable TBM, and b) Positive CSF mycobacterial culture

**O**utcome: True positive (TP), false positive (FP), true negative (TN) and false negative (FN)

**Conclusion: Insufficient evidence to recommend for or against use**

**2. How accurate are microbiological and molecular tests to diagnose TBM?**

**P**opulation: All individuals in hospital being evaluated for TBM

**I**ntervention: Each of ZN smear microscopy, Xpert, Xpert Ultra, Mycobacterial culture (MGIT, LJ, or MODS assay), Alere-LAM

**C**omparators: a) Definite or probable TBM, and b) Positive CSF mycobacterial culture

**O**utcome: True positive (TP), false positive (FP), true negative (TN) and false negative (FN)

**Conclusions:**

**a. ZN smear microscopy: Low certainty of evidence for test accuracy, weak recommendation for use in diagnosis of TBM**

**b. Xpert: High certainty of evidence for test accuracy, strong recommendation for use in diagnosis of TBM, in addition to mycobacterial culture**

**c. Xpert Ultra: Moderate certainty of evidence for test accuracy, strong recommendation for use in diagnosis of TBM, in addition to mycobacterial culture**

**d. Mycobacterial culture: Moderate certainty of evidence, strong recommendation for use in diagnosis of TBM, ideally in combination with Xpert or Xpert Ultra**

**e. Alere-Lipoarabinomannan (LAM): Insufficient evidence to recommend for or against use**

**3. How accurate is adenosine deaminase to diagnose TBM?**

**P**opulation: All individuals in hospital being evaluated for TBM

**I**ntervention: Adenosine deaminase (ADA)

**C**omparators: a) Definite or probable TBM, and b) Positive CSF mycobacterial culture

**O**utcome: True positive (TP), false positive (FP), true negative (TN) and false negative (FN)

**Conclusion: Very low certainty of evidence for test accuracy, weak recommendation for use in the diagnosis of TBM**

**4. How accurate is neuroimaging to diagnose TBM?**

**P**opulation: All individuals in hospital being evaluated for TBM or other CNS TB (e.g., spinal TB and tuberculomas)

**I**ntervention: CT or MRI brain (+/- contrast)

**C**omparators: a) Definite or probable TBM, and b) Positive CSF mycobacterial culture

**O**utcome: True positive (TP), false positive (FP), true negative (TN) and false negative (FN)

Conclusion: Insufficient evidence to recommend for or against use in diagnosis*

*Insufficient evidence to recommend for or against neuroimaging use as a diagnostic tool for TBM. However, neuroimaging may demonstrate features that increase the likelihood of a diagnosis of TBM, and neuroimaging has an important role in evaluating for intracerebral mass lesions and ensuring lumbar puncture safety (see Figure 1). Baseline neuroimaging is recommended.

Definitions: CSF=cerebrospinal fluid. CT=computed tomography. LJ=Lowenstein-Jensen. MGIT=Mycobacteria Growth Indicator Tube. MODS=Microscopic Observation Drug Susceptibility. MRI=Magnetic resonance imaging. TBM-tuberculous meningitis

## Box 2 Anti-TB chemotherapy recommendations by PICO question

**1a. Does increasing the rifampicin dose reduce mortality in adults with TBM vs. standard 10mg/kg/day dosing?**

**P**opulation: Adults in hospital requiring treatment for TBM

**I**ntervention: Rifampicin dosing of >10mg/kg/day, given orally or parenterally

**C**omparators: Rifampicin dosing of 10mg/kg/day, given orally or parenterally

**O**utcome: Mortality

Conclusion: Moderate quality of evidence, does not support increased dosing of rifampicin at 15mg/kg/day and above*

**1b. Does rifampicin dosing >20mg/kg/day reduce mortality in adults with TBM vs. standard 10mg/kg/day dosing?**

**P**opulation: Adults in hospital requiring treatment for TBM

**I**ntervention: Rifampicin dosing of >20 mg/kg/day, given orally or parenterally

**C**omparators: Rifampicin dosing of 10mg/kg/day, given orally or parenterally

**O**utcome: Mortality

Conclusion: Insufficient evidence to recommend for or against rifampicin dosing of >20mg/kg/day*

*Trial results from HARVEST (ISRCTN15668391, reporting in 2025) and INTENSE-TBM (NCT04145258, reporting in 2026) are likely to provide definitive data to address PICO questions 1a and 1b.

**2a. Does adjunctive fluoroquinolone therapy reduce mortality in adults from TBM vs. no fluoroquinolone?**

**P**opulation: Adults in hospital requiring treatment for TBM

**I**ntervention: Adjunctive fluoroquinolone

**C**omparators: Standard anti-TB chemotherapy

**O**utcome: Mortality

**Conclusions: High quality of evidence. In adults with fully drug-susceptible *M. tuberculosis*, strong recommendation against adding a fluoroquinolone to the TB regimen or using a fluoroquinolone in place of another drug in the regimen**.

**For individuals with a high probability of TBM caused by isoniazid-resistant bacteria, strong recommendation for adding a fluoroquinolone to the regimen or using a fluoroquinolone in place of isoniazid in the regimen**

**2b. Does adjunctive linezolid reduce mortality in adults from TBM vs. no linezolid?**

**P**opulation: Adults in hospital requiring treatment for TBM

**I**ntervention: Adjunctive linezolid

**C**omparators: Standard anti-TB chemotherapy

**O**utcome: Mortality

**Conclusion: Insufficient evidence to make recommendation, for or against use of linezolid**

**3. Does higher dosing, or alternative administration routes, of other TB drugs reduce mortality in adults from TBM?**

**P**opulation: Adults in hospital requiring treatment for TBM

**I**ntervention: Change in dose, route of administration or addition/substitution, of any other TB drugs

**C**omparators: Standard anti-TB chemotherapy

**O**utcome: Mortality

**Conclusion: Insufficient evidence to make recommendation, for or against higher dosing or alternative administration routes, or other TB drugs**

**4. Is treatment duration less than 12 months effective in TBM in adults?**

**P**opulation: Adults in hospital requiring treatment for TBM

**I**ntervention: Anti-TB chemotherapy duration of less than 12 months

**C**omparators: Standard anti-TB chemotherapy duration of 12 months

**O**utcome: Mortality

**Conclusion: Insufficient evidence to make a recommendation, for or against less than 12 months anti-TB chemotherapy**

**5. What is the optimal treatment for childhood TBM?**

**P**opulation: Children in hospital requiring treatment for TBM

**I**ntervention: 6 months high-dose isoniazid, high-dose rifampicin, pyrazinamide and ethionamide (6HRZEto)

**C**omparators: Standard anti-TB chemotherapy

**O**utcome: Mortality

**Conclusion: Insufficient evidence to make a recommendation for or against the shorter regimen. Either the 6-month (6H**_**15-20**_ **R**_**22.5-30**_**Z**_**35-45**_**Eto**_**17.5-22.5**_**) intensive regimen or the standard 12-month regimen can be used**.

Standard antibiotic dosing achieves subtherapeutic levels in children; please see narrative under Anti-TB chemotherapy, section 5.

## Box 3 Adjunctive therapy recommendations by PICO question

**1. Should corticosteroids be used as an adjunctive therapy in patients with TBM?**

**P**opulation: Adults and children with TBM

**I**ntervention: Anti-TB chemotherapy with adjunctive corticosteroids

**C**omparators: Anti-TB chemotherapy without adjunctive corticosteroids

**O**utcome: Mortality and morbidity

**Conclusions: High certainty of evidence, strong recommendation for use in individuals without HIV**.

**High certainty of evidence, weak recommendation for use in PLWH, therefore decision to use should be made on a case-by-case basis**

**2. What is the optimal timing of antiretroviral therapy in CNS TB?**

**P**opulation: Adults and children with TBM

**I**ntervention: Immediate ART (within 7 days of commencing anti-TB treatment)

**C**omparators: Deferred ART initiation (after 2 months of anti-TB treatment)

**O**utcome: Mortality and morbidity

**Conclusion: High certainty of evidence, weak recommendation to defer ART initiation 4-8 weeks after starting anti-TB treatment**

**3. What other adjunctive therapies can be considered for the management of TBM?**

**P**opulation: Adults and children with TBM

**I**ntervention: Adjunctive aspirin, thalidomide, infliximab, cyclophosphamide, anakinra, or interferon-gamma

**C**omparators: Anti-TB chemotherapy without above listed adjunctive agents

**O**utcome: Mortality and morbidity

**Conclusion: Insufficient evidence to make a recommendation, for or against adjunctive aspirin, thalidomide, infliximab, cyclophosphamide, anakinra, or interferon-gamma. Decision to initiate these treatments should be made on a case-by-case basis based on factors discussed in the guideline**

Definitions: ART=antiretroviral therapy. CNS=central nervous system

## Box 4 Neurocritical and neurosurgical care recommendations by PICO question

**1. Should active management (medical and/or surgical) or standard of care be used in individuals with TBM and hydrocephalus/raised ICP?**

**P**opulation: Adults or children (with or without HIV) in hospital with TBM, and hydrocephalus/raised ICP

**I**ntervention: Active medical (repeated lumbar punctures with diuretics) or surgical management (or a combination)

**C**omparators: Standard WHO therapya

**O**utcome: Mortality, and morbidity, radiological outcome, complications (including treatment failure)

**Conclusion: Insufficient evidence to make a recommendation, for or against active medical or surgical management of hydrocephalus/raised ICP**

**2. Should a VPS or ETV be used for the surgical management of hydrocephalus in patients with TBM?**

**P**opulation: Adults or children (with or without HIV) in hospital with TBM, and hydrocephalus/raised ICP

**I**ntervention: Surgical management of hydrocephalus by VPS

**C**omparators: Surgical management of hydrocephalus by ETV

**O**utcome: Mortality, morbidity, radiological outcome, complications (including treatment failure)

**Conclusion: Insufficient evidence to recommend VPS over ETV if surgical intervention is required. Discretion of the treating clinician is required**

**3. Should surgical management of tuberculomas with or without TBM occur at time of diagnosis or after medical treatment failure?**

**P**opulation: Adults or children (with or without HIV) in hospital with tuberculomas (with or without TBM)

**I**ntervention: Surgical management of tuberculomas at diagnosis

**C**omparators: Surgical management after failure of standard WHO therapies

**O**utcome: Mortality, morbidity, radiological outcome, complications (including treatment failure)

**Conclusion: Insufficient evidence to make a recommendation for or against surgical management of tuberculomas at diagnosis or after medical treatment failure**

**4. Should surgical management of TB abscesses with or without TBM occur at time of diagnosis or after medical treatment failure?**

**P**opulation: Adults or children (with or without HIV) in hospital with TB abscesses (with or without TBM)

**I**ntervention: Surgical management of TB abscesses at diagnosis

**C**omparators: Surgical management after failure of standard WHO therapies

**O**utcome: Mortality, morbidity, radiological outcome, complications (including treatment failure)

**Conclusion: Insufficient evidence to make a recommendation for or against surgical management of TB abscesses at diagnosis or after medical treatment failure**

**5. Should the management of hyponatremia in patients with TBM be based on aetiology?**

**P**opulation: Adults or children (with or without HIV) in hospital with TBM and hyponatraemia

**I**ntervention: Treatment of hyponatremia tailored by aetiology (CSW/SIADH)

**C**omparators: Treatment of hyponatremia not tailored by aetiology (CSW/SIADH)

**O**utcome: Mortality, morbidity, complications (including treatment failure)

**Conclusion: Insufficient evidence to make a recommendation for or against treatment of hyponatremia tailored by aetiology**

**6. Should all patients with TBM be assessed for clinical and sub-clinical seizures?**

**P**opulation: Adults or children (with or without HIV) in hospital with TBM

**I**ntervention: Assessed for clinical and sub-clinical seizures by EEG

**C**omparators: Not assessed for clinical and sub-clinical seizures by EEG

**O**utcome: Mortality and morbidity

**Conclusion: Insufficient evidence to make a recommendation for or against assessment for clinical and sub-clinical seizures**

^a^Standard WHO therapy refers to the current treatment regimen (2HRZE/10HR) established in 1995 and excludes streptomycin as first-line therapy.

## Key points

1. TBM diagnosis is difficult; no single test excludes TBM and anti-tuberculosis treatment is often needed based on compatible clinical features alone.
2. Xpert Ultra or Xpert, in addition to mycobacterial culture, are strongly recommended as diagnostic tests for TBM.
3. There is insufficient evidence to recommend greater than 10mg/kg/day rifampicin in adults at present; however, limited data suggests >20mg/kg/day was safe. The results of on-going phase III trials investigating higher doses (35mg/kg/day) are awaited.
4. Corticosteroids reduce mortality in TBM without HIV co-infection, and are strongly recommended as an adjunctive therapy.
5. In the absence of an effective alternative adjunctive therapy for HIV-associated TBM, and the safety and potential effectiveness of corticosteroids, corticosteroids are recommended for use in HIV-associated TBM on a case-by-case basis.
6. TBM leads to critical illness with unique neurocritical and neurosurgical considerations, yet there is little evidence guiding management strategies for these complications.
