# Supplementary material for: A Clinical Practice Guideline for Tuberculous Meningitis
Source: Lancet Infect Dis. Author manuscript; Available in PMC 2026 Feb 1. (PMC12419961; doi:10.1016/S1473-3099(25)00364-0)
Supplement: appendix [file EMS209041-supplement-appendix.pdf]

THE LANCET
Infectious Diseases

Supplementary appendix

This appendix formed part of the original submission and has been peer reviewed. We post it as supplied by the authors.

Supplement to: Donovan J, Cresswell FV, Tucker EW, et al. A clinical practice guideline for tuberculous meningitis. *Lancet Infect Dis* 2025; published online Aug 18. https://doi.org/10.1016/S1473-3099(25)00364-0.

Supplementary material list

Page 3. Supplementary table 1: Should ZN smear be used to diagnose TBM in individuals being assessed in hospital (against a standard of definite or probable TBM)?

Page 4. Supplementary table 2: Should ZN smear be used to diagnose TBM in individuals being assessed in hospital (against positive mycobacterial culture)?

Page 5. Supplementary table 3: Should Xpert be used to diagnose TBM in individuals being assessed in hospital (against a standard of definite or probable TBM)?

Page 6. Supplementary table 4: Should Xpert be used to diagnose TBM in individuals being assessed in hospital (against a standard of positive mycobacterial culture)?

Page 7. Supplementary table 5: Should Xpert Ultra be used to diagnose TBM in individuals being assessed in hospital (against a reference standard of definite or probable TBM)?

Page 8. Supplementary table 6: Should Xpert be used to diagnose TBM in individuals being assessed in hospital (against a standard of positive mycobacterial culture)?

Page 9. Supplementary table 7: Should Mycobacterial culture be used to diagnose TBM in individuals being assessed in hospital (against a reference standard of definite or probable TBM)?

Page 10. Supplementary table 8: Should Alere-Lipoarabinomannan (LAM) be used to diagnose TBM in individuals being assessed in hospital (against a reference standard of definite or probable TBM)?

Page 11. Supplementary table 9: Should Alere-Lipoarabinomannan (LAM) be used to diagnose TBM in individuals being assessed in hospital (against a standard of positive mycobacterial culture)?

Page 12. Supplementary table 10: Should Adenosine deaminase be used to diagnose TBM in individuals being assessed in hospital (against a reference standard of definite or probable TBM)?

Page 13. Supplementary table 11: Should Adenosine deaminase be used to diagnose TBM in individuals being assessed in hospital (against a reference standard of positive mycobacterial culture)?

Page 14. Supplementary table 12: Does increasing the rifampicin dosing reduce mortality in adults with TBM vs. standard 10mg/kg/day dosing?

Page 15. Supplementary table 13: Does rifampicin dosing >20mg/kg/day reduce mortality in adults with TBM vs. standard 10mg/kg/day dosing?

Page 16. Supplementary table 14: Does adjunctive fluoroquinolone reduce mortality in adults from TBM vs. no fluoroquinolone?

Page 17. Supplementary table 15: Does adjunctive linezolid reduce mortality in adults from TBM vs. no linezolid?

Page 18. Supplementary table 16: Does higher dosing, or alternative administration routes, of other TB drugs reduce mortality in adults from TBM?

Page 19. Supplementary table 17: CNS pharmacokinetics and predicted activity of second-line anti-TB drugs

Page 21. Supplementary table 18: Should corticosteroids be used as an adjunctive agent for TBM irrespective of HIV status?

Page 22. Supplementary table 19: Should corticosteroids be used as an adjunctive agent for TBM with HIV co-infection?

Page 23. Supplementary table 20: Should Immediate ART (within 7 days of commencing anti-TB treatment) be given (vs. ART deferred until 2 months after start of anti-TB treatment) in TBM with HIV co-infection?

Page 24. Supplementary table 21: What other adjunctive therapies can be considered for the management of TBM?

Page 25. Supplementary table 22: Should a VP shunt or ETV be used for the surgical management of hydrocephalus in patients with TBM?

Page 26. Supplementary data 1: Medline search strategies for each working group

Page 33. Supplementary data 2: PICO questions for which no eligible studies were returned, or the studies identified did not answer the PICO question.

Page 34. Supplementary data 3: Literature search from July 2023 to March 11th 2025.

Page 35. Supplementary data 4: Additional adjunctive therapies evaluated.

Page 36. References

Supplementary material

Supplementary table 1: Should ZN smear be used to diagnose TBM in individuals being assessed in hospital (against a standard of definite or probable TBM)?

Sensitivity	0·24 (95% CI: 0·09 to 0·52)
Specificity	0·99 (95% CI: 0·96 to 1·00)

Prevalence	50%

Outcome	№ of studies (№ of patients)	Study design	Factors that may decrease certainty of evidence					Effect per 1,000 patients tested	Test accuracy (certainty)
			Risk of bias	Indirectness	Inconsistency	Imprecision	Publication bias	pre-test probability of 50%	
**True positives** (patients with TBM)	12 studies[1–12] 927 patients	Cohort & case-control type studies	Serious[a]	Not serious	Serious[b]	Serious[c]	None	120 (45 to 260)	⊕◯◯◯ Very low
**False negatives** (patients incorrectly classified as not having TBM)								380 (240 to 455)	
**True negatives** (patients without TBM)	12 studies[1–12] 1368 patients	Cohort & case-control type studies	Serious[a]	Not serious	Serious[b]	Serious[c]	None	495 (480 to 500)	⊕◯◯◯ Very low
**False positives** (patients incorrectly classified as having TBM)								5 (0 to 20)	

Justification for downgrades: a. Justified by a small-moderate number of high and unclear judgements for 'patient selection' and 'index test'. b. Unexplained inconsistency in results; I^2 is large and therefore confidence in diagnostic estimates is lowered. ZN smear is operator dependent and therefore the process of microscopy and interpretation can vary widely across studies. c. Wide confidence intervals for pooled sensitivity and specificity. CI=confidence interval. TBM=tuberculous meningitis. ZN=Ziehl-Neelsen.

Supplementary table 2: Should ZN smear be used to diagnose TBM in individuals being assessed in hospital (against positive mycobacterial culture)?

Sensitivity	0·32 (95% CI: 0·17 to 0·51)
Specificity	0·96 (95% CI: 0·92 to 1·00)

Prevalence	50%

Outcome	№ of studies (№ of patients)	Study design	Factors that may decrease certainty of evidence					Effect per 1,000 patients tested	Test accuracy (certainty)
			Risk of bias	Indirectness	Inconsistency	Imprecision	Publication bias	pre-test probability of 50%	
**True positives** (patients with TBM)	21 studies[2,5,9,10,13–29]<br>1000 patients	Cohort & case-control type studies	Serious[a]	Not serious	Serious[b]	Serious[c]	None	160 (85 to 255)	⊕◯◯◯ Very low
**False negatives** (patients incorrectly classified as not having TBM)								340 (245 to 415)	
**True negatives** (patients without TBM)	21 studies[2,5,9,10,13–29]<br>2612 patients	Cohort & case-control type studies	Serious[a]	Not serious	Serious[b]	Serious[c]	None	480 (460 to 500)	⊕◯◯◯ Very low
**False positives** (patients incorrectly classified as having TBM)								20 (0 to 40)	

Justification for downgrades: a. Justified by a small-moderate number of high and unclear judgements for 'patient selection' and 'index test'. b. Unexplained inconsistency in results; I2 is large and therefore confidence in diagnostic estimates is lowered. ZN smear is operator dependent and therefore the process of microscopy and interpretation can vary widely across studies. c. Wide confidence intervals for pooled sensitivity and specificity. CI=confidence interval. TBM=tuberculous meningitis. ZN=Ziehl-Neelsen.

Supplementary table 3: Should Xpert be used to diagnose TBM in individuals being assessed in hospital (against a standard of definite or probable TBM)?

Sensitivity	0·43 (95% CI: 0·38 to 0·49)
Specificity	0·99 (95% CI: 0·97 to 0·99)

Prevalence	50%

Outcome	№ of studies (№ of patients)	Study design	Factors that may decrease certainty of evidence					Effect per 1,000 patients tested	Test accuracy (certainty)
			Risk of bias	Indirectness	Inconsistency	Imprecision	Publication bias	pre-test probability of 50%	
**True positives** (patients with TBM)	24 studies[1–4,6–10,12,19,30–42] 1480 patients	Cohort & case-control type studies	Not serious	Not serious	Not serious	Not serious	None	215 (190 to 245)	⊕⊕⊕⊕ High
**False negatives** (patients incorrectly classified as not having TBM)								285 (255 to 310)	
**True negatives** (patients without TBM)	24 studies[1–4,6–10,12,19,30–42] 2059 patients	Cohort & case-control type studies	Not serious	Not serious	Not serious	Not serious	None	495 (485 to 495)	⊕⊕⊕⊕ High
**False positives** (patients incorrectly classified as having TBM)								5 (5 to 15)	

CI=confidence interval. TBM=tuberculous meningitis.

Supplementary table 4: Should Xpert be used to diagnose TBM in individuals being assessed in hospital (against a standard of positive mycobacterial culture)?

Sensitivity	0·59 (95% CI: 0·50 to 0·67)
Specificity	0·95 (95% CI: 0·93 to 0·97)

Prevalence	50%

Outcome	№ of studies (№ of patients)	Study design	Factors that may decrease certainty of evidence					Effect per 1,000 patients tested	Test accuracy (certainty)
			Risk of bias	Indirectness	Inconsistency	Imprecision	Publication bias	pre-test probability of 50%	
**True positives** (patients with TBM)	21 studies[2,3,8–10,14,17–19,30–34,37,42–47] 640 patients	Cohort & case-control type studies	Not serious	Not serious	Not serious	Not serious	None	295 (250 to 335)	⊕⊕⊕⊕ High
**False negatives** (patients incorrectly classified as not having TBM)								205 (165 to 250)	
**True negatives** (patients without TBM)	21 studies[2,3,8–10,14,17–19,30–34,37,42–47] 3068 patients	Cohort & case-control type studies	Not serious	Not serious	Not serious	Not serious	None	475 (465 to 485)	⊕⊕⊕⊕ High
**False positives** (patients incorrectly classified as having TBM)								25 (15 to 35)	

CI=confidence interval. TBM=tuberculous meningitis.

Supplementary table 5: Should Xpert Ultra be used to diagnose TBM in individuals being assessed in hospital (against a reference standard of definite or probable TBM)?

Sensitivity	0·65 (95% CI: 0·59 to 0·71)
Specificity	0·99 (95% CI: 0·96 to 0·99)

Prevalence	50%

Outcome	№ of studies (№ of patients)	Study design	Factors that may decrease certainty of evidence					Effect per 1,000 patients tested	Test accuracy (certainty)
			Risk of bias	Indirectness	Inconsistency	Imprecision	Publication bias	pre-test probability of 50%	
**True positives** (patients with TBM)	8 studies[3,34,36,37,40,48–50] 684 patients	Cohort & case-control type studies	Serious[a]	Not serious	Not serious	Not serious	None	325 (295 to 355)	⊕⊕⊕◯ Moderate
**False negatives** (patients incorrectly classified as not having TBM)								175 (145 to 205)	
**True negatives** (patients without TBM)	8 studies[3,34,36,37,40,48–50] 591 patients	Cohort & case-control type studies	Serious[a]	Not serious	Not serious	Not serious	None	495 (480 to 495)	⊕⊕⊕◯ Moderate
**False positives** (patients incorrectly classified as having TBM)								5 (5 to 20)	

Justification for downgrades: a. Justified by small-moderate number of high and unclear judgements for 'patient selection' and 'index test'. CI=confidence interval. TBM=tuberculous meningitis.

Supplementary table 6: Should Xpert Ultra be used to diagnose TBM in individuals being assessed in hospital (against a standard of positive mycobacterial culture)?

Sensitivity	0·83 (95% CI: 0·76 to 0·89)
Specificity	0·79 (95% CI: 0·63 to 0·90)

Prevalence	50%

Outcome	№ of studies (№ of patients)	Study design	Factors that may decrease certainty of evidence					Effect per 1,000 patients tested	Test accuracy (certainty)
			Risk of bias	Indirectness	Inconsistency	Imprecision	Publication bias	pre-test probability of 50%	
**True positives** (patients with TBM)	8 studies[3,34,37,43,48–51] 140 patients	Cohort & case-control type studies	Serious[a]	Not serious	Not serious	Not serious	None	415 (380 to 445)	⊕⊕⊕◯ Moderate
**False negatives** (patients incorrectly classified as not having TBM)								85 (55 to 120)	
**True negatives** (patients without TBM)	8 studies[3,34,37,43,48–51] 748 patients	Cohort & case-control type studies	Serious[a]	Not serious	Not serious	Not serious	None	395 (315 to 450)	⊕⊕⊕◯ Moderate
**False positives** (patients incorrectly classified as having TBM)								105 (50 to 185)	

Justification for downgrades: a. Justified by small-moderate number of high and unclear judgements for 'patient selection' and 'index test'. CI=confidence interval. TBM=tuberculous meningitis.

Supplementary table 7: Should Mycobacterial culture be used to diagnose TBM in individuals being assessed in hospital (against a reference standard of definite or probable TBM)?

Sensitivity	0·33 (95% CI: 0·23 to 0·44)
Specificity	0·99 (95% CI: 0·99 to 1·00)

Prevalence	50%

Outcome	№ of studies (№ of patients)	Study design	Factors that may decrease certainty of evidence					Effect per 1,000 patients tested	Test accuracy (certainty)
			Risk of bias	Indirectness	Inconsistency	Imprecision	Publication bias	pre-test probability of 50%	
**True positives** (patients with TBM)	14 studies[1–4,8,9,30,34–37,40,50,52] 1004 patients	Cohort & case-control type studies	Not serious[a]	Not serious	Serious	Not serious	none	165 (115 to 220)	⊕⊕⊕◯ Moderate
**False negatives** (patients incorrectly classified as not having TBM)								335 (280 to 385)	
**True negatives** (patients without TBM)	14 studies[1–4,8,9,30,34–37,40,50,52] 1536 patients	Cohort & case-control type studies	Not serious[a]	Not serious	Serious	Not serious	none	495 (495 to 500)	⊕⊕⊕◯ Moderate
**False positives** (patients incorrectly classified as having TBM)								5 (0 to 5)	

Justification for downgrades: a. Unexplained inconsistency in results which may be due to different culturing processes and cerebrospinal fluid volume tested, and varying culture media. CI=confidence interval. TBM=tuberculous meningitis.

Supplementary table 8: Should Alere-Lipoarabinomannan (LAM) be used to diagnose TBM in individuals being assessed in hospital (against a reference standard of definite or probable TBM)?

Sensitivity	0·24 (95% CI: 0·07 to 0·50)
Specificity	0·95 (95% CI: 0·84 to 0·99)

Prevalence	50%

Outcome	№ of studies (№ of patients)	Study design	Factors that may decrease certainty of evidence					Effect per 1,000 patients tested	Test accuracy (certainty)
			Risk of bias	Indirectness	Inconsistency	Imprecision	Publication bias	pre-test probability of 50%	
**True positives** (patients with TBM)	1 study[53] 17 patients	Cross-sectional (cohort type accuracy study)	Serious[a]	Not serious	Not assessable	Very serious	Not assessable	120 (35 to 250)	⊕◯◯◯ Very low[b]
**False negatives** (patients incorrectly classified as not having TBM)								380 (250 to 465)	
**True negatives** (patients without TBM)	1 study[53] 42 patients	Cross-sectional (cohort type accuracy study)	Serious[a]	Not serious	Not assessable	Very serious	Not assessable	475 (420 to 495)	⊕◯◯◯ Very low[b]
**False positives** (patients incorrectly classified as having TBM)								25 (5 to 80)	

Justification for downgrades: a. Justified by unclear judgements for all risk of bias domains. b. Single study limits assessment of inconsistency and publication bias. Overall test accuracy given as very low. CI=confidence interval. TBM=tuberculous meningitis.

Supplementary table 9: Should Alere-Lipoarabinomannan (LAM) be used to diagnose TBM in individuals being assessed in hospital (against a standard of positive mycobacterial culture)?

Sensitivity	0·22 (95% CI: 0·14 to 0·31)
Specificity	0·94 (95% CI: 0·92 to 0·96)

Prevalence	50%

Outcome	№ of studies (№ of patients)	Study design	Factors that may decrease certainty of evidence					Effect per 1,000 patients tested	Test accuracy (certainty)
			Risk of bias	Indirectness	Inconsistency	Imprecision	Publication bias	pre-test probability of 50%	
**True positives** (patients with TBM)	1 study[54] 105 patients	Cross-sectional (cohort type accuracy study)	Serious[a]	Not serious	Not assessable	Very serious	Not assessable	110 (70 to 155)	⨁◯◯◯ Very low[b]
**False negatives** (patients incorrectly classified as not having TBM)								390 (345 to 430)	
**True negatives** (patients without TBM)	1 study[54] 424 patients	Cross-sectional (cohort type accuracy study)	Serious[a]	Not serious	Not assessable	Very serious	Not assessable	470 (460 to 480)	⨁◯◯◯ Very low[b]
**False positives** (patients incorrectly classified as having TBM)								30 (20 to 40)	

Justification for downgrades: a. Justified by high risk of bias for patient selection domain. b. Single study limits assessment of inconsistency and publication bias. Overall test accuracy given as very low. CI=confidence interval. TBM=tuberculous meningitis.

Supplementary table 10: Should Adenosine deaminase be used to diagnose TBM in individuals being assessed in hospital (against a reference standard of definite or probable TBM)?

Sensitivity	0·83 (95% CI: 0·74 to 0·90)
Specificity	0·91 (95% CI: 0·85 to 0·94)

Prevalence	50%

Outcome	№ of studies (№ of patients)	Study design	Factors that may decrease certainty of evidence					Effect per 1,000 patients tested	Test accuracy (certainty)
			Risk of bias	Indirectness	Inconsistency	Imprecision	Publication bias	pre-test probability of 50%	
**True positives** (patients with tuberculous meningitis)	17 studies[55–71] 471 patients	Cohort & case-control type studies	Serious[a]	Not serious	Serious[b]	Serious	None	417 (371 to 450)	⊕◯◯◯ Very low[a,b]
**False negatives** (patients incorrectly classified as not having tuberculous meningitis)								83 (50 to 129)	
**True negatives** (patients without tuberculous meningitis)	17 studies[55–71] 1451 patients	Cohort & case-control type studies	Serious[a]	Not serious	Serious[b]	Serious	None	454 (427 to 472)	⊕◯◯◯ Very low[a,b]
**False positives** (patients incorrectly classified as having tuberculous meningitis)								46 (28 to 73)	

Justification for downgrades: a. Justified by large number of high and unclear judgements for ‘patient selection’ and ‘index test’. b. Unexplained inconsistency in results (differing sensitivity/specificity and confidence intervals when adenosine deaminase cut-offs are the same or similar). CI=confidence interval. TBM=tuberculous meningitis.

Supplementary table 11: Should Adenosine deaminase be used to diagnose TBM in individuals being assessed in hospital (against a reference standard of positive mycobacterial culture)?

Sensitivity	0·79 (95% CI: 0·69 to 0·86)
Specificity	0·97 (95% CI: 0·92 to 0·99)

Prevalence	50%

Outcome	№ of studies (№ of patients)	Study design	Factors that may decrease certainty of evidence					Effect per 1,000 patients tested	Test accuracy (certainty)
			Risk of bias	Indirectness	Inconsistency	Imprecision	Publication bias	pre-test probability of 50%	
**True positives** (patients with tuberculous meningitis)	4 studies[61,71–73] 85 patients	Cohort & case-control type studies	Serious[a]	Not serious	Serious[b]	Serious	none	394 (344 to 432)	⊕◯◯◯ Very low[a,b]
**False negatives** (patients incorrectly classified as not having tuberculous meningitis)								106 (68 to 156)	
**True negatives** (patients without tuberculous meningitis)	4 studies[61,71–73] 219 patients	Cohort & case-control type studies	Serious[a]	Not serious	Serious[b]	Serious	none	485 (459 to 495)	⊕◯◯◯ Very low[a,b]
**False positives** (patients incorrectly classified as having tuberculous meningitis)								15 (5 to 41)	

Justification for downgrades: a. Justified by large number of high and unclear judgements for ‘patient selection’ and ‘index test’. b. Unexplained inconsistency in results (differing sensitivity/specificity and confidence intervals when adenosine deaminase cut-offs are the same or similar). CI=confidence interval. TBM=tuberculous meningitis.

Supplementary table 12: Does increasing the rifampicin dosing reduce mortality in adults in TBM vs. standard 10mg/kg/day dosing?

Certainty assessment							№ of patients		Effect		Certainty
№ of studies	Study design	Risk of bias	Inconsistency	Indirectness	Imprecision	Other considerations	Any elevated dose of rifampicin	Normal dose rifampicin	Relative (95% CI)	Absolute (95% CI)	
**Death (follow-up: range 3 months to 12 months)**											
5	Randomised trials[74–78]	Not serious	Serious[a]	Not serious	Serious[b]	None	164/566 (29·0%)	158/521 (30·3%)	**OR 0·91** (0·56 to 1·46)	**20 fewer per 1,000** (from 107 fewer to 85 more)	⨁⨁◯◯ Low[a,b]

Justification for downgrades: a. Inconsistent results from African and Asian studies. b. Very wide confidence intervals in individual studies and in the main analysis. CI=confidence interval. OR=odds ratio. TBM=tuberculous meningitis.

Supplementary table 13: Does rifampicin dosing >20mg/kg/day reduce mortality in adults in TBM vs. standard 10mg/kg/day dosing?

Certainty assessment							№ of patients		Effect		Certainty
№ of studies	Study design	Risk of bias	Inconsistency	Indirectness	Imprecision	Other considerations	Rifampicin at >20mg/kg	Rifampicin at normal dosage	Relative (95% CI)	Absolute (95% CI)	
**Death (follow-up: range 3 months to 12 months)**											
4	Randomised trials[74,76–78]	Not serious	Serious[a]	Not serious[b]	Serious[c]	None	42/138 (30·4%)	44/112 (39·3%)	**OR 0·71** (0·26 to 1·92)	**78 fewer per 1,000** (from 249 fewer to 161 more)	⊕⊕◯◯ Low

Justification for downgrades: a. Inconsistent African and Asian results. b. Inconsistent dosing between studies. c. Very wide confidence intervals in individual studies and in the meta-analysis. CI=confidence interval. OR=odds ratio. TBM=tuberculous meningitis.

Supplementary table 14: Does adjunctive fluoroquinolone reduce mortality in adults from TBM vs. no fluoroquinolone?

Certainty assessment							№ of patients		Effect		Certainty
№ of studies	Study design	Risk of bias	Inconsistency	Indirectness	Imprecision	Other considerations	A fluoroquinolone	No fluoroquinolone	Relative (95% CI)	Absolute (95% CI)	
**Death (follow-up: range 3 months to 12 months)**											
5	Randomised trials [75,76,79–81]	Not serious	Not serious	Not serious	Not serious	None	161/581 (27·7%)	157/534 (29·4%)	**OR 0·86** (0·51 to 1·**45**)	**30 fewer per 1,000** (from 119 fewer to 82 more)	⨁⨁⨁⨁ High

CI=confidence interval. OR=odds ratio. TBM=tuberculous meningitis.

Supplementary table 15: Does adjunctive linezolid reduce mortality in adults from TBM vs. no linezolid?

Certainty assessment							№ of patients		Effect		Certainty
№ of studies	Study design	Risk of bias	Inconsistency	Indirectness	Imprecision	Other considerations	Linezolid	no linezolid	Relative (95% CI)	Absolute (95% CI)	
**Death (follow-up: range 3 months to 12 months)**											
3	Randomised trials[78,82,83]	Not serious	Not serious	Not serious	Very serious[a]	None	13/65 (12·0%)	16/54 (29·6%)	**OR 0·63** (0·17 to 2.28)	**87 fewer per 1,000** (from 229 fewer to 193 more)	⊕⊕◯◯ Low

Justification for downgrades: a. very small studies with very wide confidence intervals. CI=confidence interval. OR=odds ratio. TBM=tuberculous meningitis.

Supplementary table 16: Does higher dosing, or alternative administration routes, of other TB drugs reduce mortality in adults from TBM?

Certainty assessment							№ of patients		Effect		Certainty
№ of studies	Study design	Risk of bias	Inconsistency	Indirectness	Imprecision	Other considerations	Does higher dosing, or alternative administration routes, of other TB drugs	standard dosing or administration routes	Relative (95% CI)	Absolute (95% CI)	
**Mortality (follow-up: 6 months)**											
1	Randomised trial [84]	Serious[a]	Serious[b,c]	Not serious	Serious[d]	None	9/23 (39·1%)	22/31 (71·0%)	**OR 3·80** (1·21 to 11·90)	**193 more per 1,000** (from 38 more to 257 more)	⊕○○○ Very low

This study compares high dose isoniazid and ethambutol vs. standard of care for TBM in adults. Justification for downgrades: a. Open label trial. b. Only one study, therefore difficult to generalise. c. Mortality in the control arm was unusually high (71%) and mortality in the intervention arm was what is expected with standard therapy (39%). d. Small study size and wide confidence intervals. CI=confidence interval. OR=odds ratio. TBM=tuberculous meningitis.

Drug	Form	Oral bio-availability (%)	Food effect	Plasma protein binding (%)	CSF penetration clinical studies (%)	Animal data Brain/CSF penetration Bacterial killing	Metabolism/ Elimination	Half-life	Relevant interactions	Toxicity	Anticipated utility in DR-TBM
**Second-line**											
Streptomycin[85]	IV; IM	~0	N/A	35	Low	x	Mostly unmetabolised<br><br>Excretion: Urine	5-6 hr	Loop diuretics	Nephrotoxicity (kanamycin = amikacin > streptomycin); ototoxicity; vestibular toxicity; electrolyte abnormalities (hypokalaemia, hypocalcaemia, hypomagnesaemia)	Low
Kanamycin	IV; IM	~0	N/A	~0	10-20	ND	Mostly unmetabolised<br><br>Excretion: urine	2.5 hr	Loop diuretics	Similar to streptomycin	Low
Amikacin	IV; IM	~0	N/A	0-11	10-20	ND	Mostly unmetabolised<br><br>Excretion: Urine	2-3 hr	Loop diuretics	Similar to streptomycin	Low
Capreomycin	IV; IM	~0	N/A	0-10	x	ND	Mostly unmetabolised<br><br>Excretion: Urine	5.2-6.8 hr	Loop diuretics	Similar to streptomycin	Low
Levofloxacin	PO; IV	~100	None	24-38	70-80	ND	Mostly unmetabolised (>95% excreted unchanged)<br><br>Excretion: urine (87%)	6-8 hr	QT prolongation	Nausea; bloating; headache; dizziness; insomnia; tremulousness; tendon rupture; arthralgias; QTc prolongation; hypo/hyperglycaemia; hepatotoxicity	High
Moxifloxacin[86]	PO; IV	90	None	50	70-80	Mean brain/plasma ratio of 23%	Hepatic (45% excreted unchanged)<br><br>Excretion: urine (~70%); faeces (~30%)	11.5-15.6 hr	QT prolongation	Similar to levofloxacin	High
Ethionamide[87]	PO	~100	None	~30	80-90	ND	Hepatic (<1% excreted unchanged)<br><br>Excretion: urine	2-3 hr	Isoniazid; cycloserine	Gastrointestinal upset and anorexia; metallic taste; hepatotoxicity; gynaecomastia; hair loss; acne; impotence; menstrual irregularity; reversible hypothyroidism; neurotoxicity	High
Cycloserine[88–90]	PO	65-90	Slight decrease	~0	50-60	ND	Mostly unmetabolised<br><br>Excretion: Urine	10-14 hr	Isoniazid; ethionamide	CNS toxicity (inability to concentrate; lethargy; seizures; depression; psychosis; suicidal ideation); peripheral neuropathy; skin changes (lichenoid eruptions; Stevens-Johnson syndrome)	Moderate
Terizidone[91]	PO	~70	Unknown	Unknown	69	Unknown	Hydrolysed<br><br>Excretion: Urine	12-18 hr	Isoniazid; ethionamide	CNS toxicity (depression, anxiety, psychosis, seizures); peripheral neuropathy; skin reactions; gastrointestinal intolerance	Moderate
Linezolid[86,92–94]	PO; IV	~100	-23% with high-fat meals	31	30	$AUC_{0\text{-}60min}$ brain/plasma ratio of 28% (PET studies)	Mostly unmetabolised<br><br>Excretion: Urine	4.5-5.5 hr	Serotonergic agents	Myelosuppression; diarrhoea; nausea; optic/peripheral neuropathy; lactic acidosis.	Moderate to high

Bedaquiline[86,95,96]	PO	Unknown	Increase	>99	Likely poor (limited data)	AUC_{0-48h} brain/plasma ratio of 0·21 (PET studies); CSF levels undetectable; no additive bacterial killing with addition of bedaquiline	Hepatic oxidation (<1% excreted unchanged)<br><br>Excretion: urine	5.5 months	P450 inducers/ inhibitors QTc prolongation	Arthralgia; dizziness; headache; hyperuricaemia; insomnia; myalgia; nausea; pruritus; vomiting; QTc prolongation	Low
Delamanid[88,97,98]	PO	25-47	Increase	>99	Low / undetectable CSF levels	Low CSF levels	Albumin > hepatic metabolism<br><br>Excretion: stool; urine (<5%)	30-38 hr	P450 inducers/ inhibitors QTc prolongation	Headache; dizziness; nausea; paraesthesia; QTc prolongation	Low
Pretomanid[86,99–101]	PO	Good bioavailability, increasing with increasing dose	Increase	93	$AUC_{0-60min}$ brain/plasma ratio of 225% (PET studies)	$AUC_{0-60min}$ brain/plasma ratio of 141% (PET studies); excellent bacterial killing especially when combined with pyrazinamide	Urinary and hepatic	16-20 hr	Possible QTc prolongation	Gastrointestinal	High
Clofazamine[88,93,98,102–104]	PO	~50	Increase	>90%	Undetectable CSF levels	Mean brain/plasma ratio of 21·4-35·1%	Dehalogenation and glucoronidation	25 days	QTc prolongation	Skin discolouration. QTc prolongation	Low

Supplementary table 17: CNS pharmacokinetics and predicted activity of second-line anti-TB drugs

CNS=central nervous system. DR=drug resistant. IM=intramuscular. IV=intravenous. N/A=not applicable. ND=no data. PET=Positron Emission Tomography. PO=oral administration.

Supplementary table 18: Should corticosteroids be used as an adjunctive agent for TBM irrespective of HIV status?

Certainty assessment							№ of patients		Effect		Certainty
№ of studies	Study design	Risk of bias	Inconsistency	Indirectness	Imprecision	Other considerations	corticosteroids	no corticosteroids	Relative (95% CI)	Absolute (95% CI)	
**Mortality (follow-up: range 9 months to 12 months)**											
9	Randomised trials,[105–113]	Not serious	Not serious	Not serious	Not serious	None	316/910 (34·7%)	367/860 (42·7%)	**RR 0·81** (0·72-0·92)	**79 fewer per 1,000** (from 34 fewer to 125 fewer)	⊕⊕⊕⊕ High
**Morbidity** as defined by modified Rankin Scale 3-5 (compared to 0-2) amongst survivors with follow-up from 9 to 12 months)											
8	Randomised trials,[105–112]	Not serious	Serious	Not serious	Not serious	None	102/589 (17·3%)	100/490 (20·4%)	**RR 0·85** (0·66-1·09)	**31 fewer per 1,000 survivors** (from 78 fewer to 16 more)	⊕⊕⊕O Moderate

CI=confidence interval. OR=odds ratio. RR=risk ratio. TBM=tuberculous meningitis.

Supplementary table 19: Should corticosteroids be used as an adjunctive agent for TBM with HIV co-infection?

Certainty assessment							№ of patients		Effect		Certainty
№ of studies	Study design	Risk of bias	Inconsistency	Indirectness	Imprecision	Other considerations	Corticosteroids	no corticosteroids	Relative (95% CI)	Absolute (95% CI)	
**Mortality (follow-up: range 9 months to 12 months)**											
2	Randomised trials[111,112]	Not serious	Not serious	Not serious	Not serious	None	143/307 (46·6%)	163/311 (52·4%)	**RR 0·90** (0·77 to 1·05)	**52 fewer per 1,000** (from 121 fewer to 26 more)	⊕⊕⊕⊕ High
**Morbidity** as defined by modified Rankin Scale 3-5 (compared to 0-2) amongst survivors with follow-up from 9 to 12 months)											
2	Randomised trials[111,112]	Not serious	Not serious	Not serious	Not serious	None	15/147 (10·2%)	10/131 (7·6%)	**RR 1·34** (0·62 to 2·87)	**25 more per 1,000** (from 41 fewer to 93 more)	⊕⊕⊕⊕ High

CI=confidence interval. OR=odds ratio. RR=risk ratio. TBM=tuberculous meningitis.

Supplementary table 20: Should immediate ART (within 7 days of commencing anti-TB treatment) be given (vs. ART deferred until 2 months after start of anti-TB treatment) in TBM with HIV co-infection?

Certainty assessment							№ of patients		Effect		Certainty
№ of studies	Study design	Risk of bias	Inconsistency	Indirectness	Imprecision	Other considerations	immediate	deferred ART	Relative (95% CI)	Absolute (95% CI)	
**Mortality (follow-up: 9 months)**											
1	Randomised trial[114]	Not serious	Not serious	Not serious	Not serious	None	76/127 (59·8%)	70/126 (55·6%)	**HR 1·12** (0·81 to 1·55)	**41 more per 1,000** (from 74 fewer to 160 more)	⊕⊕⊕⊕ High
**Grade 3 or 4 adverse events within first 2 months (follow-up: 2 months)**											
1	Randomised trial[114]	Not serious	Not serious	Not serious	Not serious	None	109/127 (85·8%)	95/126 (75·4%)	**RR 1·14** (1·01 to 1·29)	**106 more per 1,000** (from 8 more to 219 more)	⊕⊕⊕⊕ High
**Grade 4 adverse events within first 2 months (follow-up: 2 months)**											
1	Randomised trial[114]	Not serious	Not serious	Not serious	Not serious	None	77/127 (60·6%)	59/126 (46·8%)	**RR 1·29** (1·03 to 1·63)	**136 more per 1,000** (from 14 more to 295 more)	⊕⊕⊕⊕ High

ART=antiretroviral therapy. CI=confidence interval. HR=hazard ratio. OR=odds ratio. RR=risk ratio. TBM=tuberculous meningitis.

Supplementary table 21: What other adjunctive therapies can be considered for the management of TBM?

Certainty assessment							№ of patients		Effect		Certainty
№ of studies	Study design	Risk of bias	Inconsistency	Indirectness	Imprecision	Other considerations	aspirin	no aspirin	Relative (95% CI)	Absolute (95% CI)	
4	Randomised trials[78,115–117]	Not serious	Serious	Serious	Not serious	None	25/250 (10·0%)	35/170 (20·6%)	**RR 0·59** (0·34 to 1·02)	**84 fewer per 1,000** (from 136 fewer to 5 more)	⊕⊕OO

Data shown for the effect of aspirin mortality. Adult (N=3) and paediatric (N=1) trials pooled. Additional searches for thalidomide, infliximab, cyclophosphamide, anakinra, or interferon-gamma. Data for these studies are described in supplementary data. CI=confidence interval. RR=risk ratio. TBM=tuberculous meningitis.

Supplementary table 22: Should a VP shunt or ETV be used for the surgical management of hydrocephalus in patients with TBM?

Certainty assessment						№ of patients		Effect	Certainty
№ of studies	Study design	Risk of bias	Inconsistency	Indirectness	Imprecision	VP shunt	ETV	Relative (95% CI)	
**Mortality (follow-up: range 5-6 months)**									
2	Randomised trials[118,119]	Serious[a]	Not serious	Serious[b]	Serious[c]	4/50 (8·0%)	4/50 (8·0%)	**OR 1·00** (0·13 to 7·70)	⊕◯◯◯ Very low
**Success - no further intervention needed (follow-up: range 5-6 months)**									
2	Randomised trials[118,119]	Serious[a]	Serious[d]	Serious[b]	Not serious	29/50 (58·0%)	27/50 (54·0%)	**OR 0·85** (0·38 to 1·89)<br>**RR 0·96** (0·68 to 1·35)	⊕◯◯◯ Very low

[a]Risk of bias was low using RoB2 but downgraded due to small sample size. [b]Only paediatric patients (< 18 years), unclear HIV status and large proportion of patients were only probable TBM (by definition). [c]Wide confidence interval and small sample size. [d]Definition of "success" was inferred from the definition of failure which was the need for another surgical intervention. CI=confidence interval. OR=odds ratio. RR=risk ratio. TBM=tuberculous meningitis.

Supplementary data 1

Search strategies

Medline search strategies for each working group are provided below for searches performed on March 11th 2025. Search strategies for all databases will be provided with any subsequently published systematic reviews associated with the guideline.

Diagnosis

1	Tuberculosis, Meningeal/	7606
2	((Tubercul* or TB) adj4 (meningitis or meningeal or meninges or meningitic or pachymening* or meningoencepha*)).tw,kf.	8600
3	Tuberculosis, Central Nervous System/	488
4	tuberculoma, intracranial/	695
5	tuberculoma/ and (intracranial* or cranial* or brain or midbrain or spinal cord* or cereb* or cns or central nervous or nervous system or pituitary or radiculomyelitis or arachnoiditis or myeloradiculopathy or neuro* or nerve*).tw,kf,hw.	906
6	((intracranial* or cranial* or brain or midbrain or spinal cord* or cereb* or cns or central nervous or nervous system or pituitary or radiculomyelitis or arachnoiditis or myeloradiculopathy or neuro* or nerve*) adj6 (tubercul* or TB)).tw,kf,hw.	7130
7	(Arachnoiditis/ or Meningoencephalitis/) and (tubercul* or TB).mp.	521
8	TBM.tw,kf.	2712
9	Neurotuberculosis.tw,kf.	167
10	or/1-9	16202
11	Cerebrospinal Fluid/	19778
12	Cerebrospinal Fluid Proteins/	4418
13	(CSF or cerebrospinal fluid* or spinal fluid*).tw,kf.	188298
14	Spinal Puncture/	6982
15	(lumber puncture* or spinal puncture* or spinal tap or spinal taps).tw,kf.	1147
16	cf.fs.	54793
17	11 or 12 or 13 or 14 or 15 or 16	208320
18	exp Leukocytes/ or exp Leukocyte Count/ or Lymphocytosis/	902757
19	(lymphocyt* or leu?ocyte* or white blood cell* or pleocytosis or pleiocytosis or WBC count* or neutrophil*).tw,kf.	791389
20	Glucose/	182197
21	glucose.tw,kf,nm.	712860
22	Hypoglycorrhachia.tw,kf.	327
23	exp Lactic Acid/ or Lactates/	78942
24	(lactate* or lactic acid*).tw,kf,nm.	235917
25	Cerebrospinal Fluid Proteins/	4418
26	(protein* or hyperproteinorrhachia).tw,kf,nm.	5621152
27	(biochemistry or fluid chemistry or csf chemistry).mp.	71008
28	conventional bacteriology.mp.	35
29	(CSF parameter* or fluid* parameter*).mp.	1011
30	or/18-29	7140003
31	10 and 17 and 30 [question 1]	1144
32	(mZN or ZN or Ziehl-Neelsen* or Xpert* or GeneXpert* or MGIT* or Mycobacteria growth indicator* or Lowenstein-Jensen or LJ or MODS).mp.	113887
33	(microscopic observation adj2 drug susceptibility).mp.	124
34	(Lipoarabinomannan or lam or alere* or fuji or tblam* or tb lam*).mp.	12281
35	exp Nucleic Acid Amplification Techniques/	483244

36	(nucleic acid amplification or NAAT or NAA test* or polymerase chain reaction or pcr).tw,kf.	829204
37	((csf or cerebrospinal fluid* or spinal fluid*) adj2 microscopy).mp.	89
38	(Acid-fast or AFB).mp.	12145
39	Molecular Diagnostic Techniques/	14744
40	((culture or smear) and (sensitiv* or specificity)).tw,kf.	81659
41	or/32-40	1234397
42	10 and 41 [ques 2]	1246
1	Tuberculosis, Meningeal/	7606
2	((Tubercul* or TB) adj4 (meningitis or meningeal or meninges or meningitic or pachymening* or meningoencepha*)).tw,kf.	8600
3	Tuberculosis, Central Nervous System/	488
4	tuberculoma, intracranial/	695
5	tuberculoma/ and (intracranial* or cranial* or brain or midbrain or spinal cord* or cereb* or cns or central nervous or nervous system or pituitary or radiculomyelitis or arachnoiditis or myeloradiculopathy or neuro* or nerve*).tw,kf,hw.	906
6	((intracranial* or cranial* or brain or midbrain or spinal cord* or cereb* or cns or central nervous or nervous system or pituitary or radiculomyelitis or arachnoiditis or myeloradiculopathy or neuro* or nerve*) adj6 (tubercul* or TB)).tw,kf,hw.	7130
7	(Arachnoiditis/ or Meningoencephalitis/) and (tubercul* or TB).mp.	521
8	TBM.tw,kf.	2712
9	Neurotuberculosis.tw,kf.	167
10	1 or 2 or 3 or 4 or 5 or 6 or 7 or 8 or 9	16202
11	Adenosine Deaminase/	7519
12	Adenosine/ or Aminohydrolases/	37201
13	limit 12 to yr="1972 - 1974"	1388
14	(Adenosine adj2 (aminohydrolase or deaminase)).mp.	11835
15	(IGRA or interferon gamma or Quantiferon* or Tspot* or T-spot*).mp.	106413
16	(Interferon* adj2 release assay*).mp.	2861
17	(IFN* adj2 release assay*).mp.	554
18	(ELISPOT* or ELISA or enzyme-linked immune absorbent or enzyme-linked immunospot or enzyme-linked immunosorbent or enzyme Immunoassay).mp.	367214
19	Interferon-gamma Release Tests/	2141
20	enzyme-linked immunosorbent assay/ or enzyme-linked immunospot assay/	157414
21	11 or 13 or 14 or 15 or 16 or 17 or 18 or 19 or 20	472201
22	10 and 21 [question 3]	651
23	exp Magnetic Resonance Spectroscopy/	226705
24	(magnetic resonance or mr spectroscop* or nmr spectroscop*).tw,kf.	535779
25	Magnetic Resonance Imaging/	505799
26	(fmri or mri or MR imag* or MR scan* or mris).tw,kf.	460223
27	exp tomography, emission-computed/ or exp tomography, x-ray computed/	609953
28	(comput* tomograph* or CT scan*).tw,kf.	501909
29	exp Neuroimaging/	205821
30	(neuro-imaging or neuroimaging or brain imaging).tw,kf.	93559
31	23 or 24 or 25 or 26 or 27 or 28 or 29 or 30	1886732
32	10 and 31 [Qu 4 neuroimaging]	3287
33	Tuberculosis, Meningeal/di	2067
34	Tuberculosis, Central Nervous System/di	239
35	tuberculoma, intracranial/di	391
36	exp "Sensitivity and Specificity"/	679385
37	(sensitiv* or specificity).tw,kf.	2085259

38	(predictive adj4 value$).tw,kf.	157595
39	diagnostic errors/ or false negative reactions/ or false positive reactions/	79331
40	(false positive* or true positive* or false negative* or true negative*).tw,kf.	101383
41	roc.tw,kf.	103908
42	accura*.tw,kf.	1231438
43	"diagnos*".m_titl.	741888
44	diagnos*.kf.	199042
45	(diagnos* adj4 (scan or mri or mr scan* or magnetic resonance or imag* or neuroimag* or ct or comput* tomograph* or fmri or MR imag* or mris)).ab.	124709
46	Tuberculosis, Meningeal/dg	322
47	Tuberculosis, Central Nervous System/dg	82
48	tuberculoma, intracranial/dg	173
49	((pre-test or pretest) adj probability).tw.	3119
50	post-test probability.tw.	793
51	likelihood ratio$.tw.	21555
52	33 or 34 or 35 or 36 or 37 or 38 or 39 or 40 or 41 or 42 or 43 or 44 or 45 or 46 or 47 or 48 or 49 or 50 or 51	4276973
53	32 and 52	1715

Anti-TB chemotherapy

1	Tuberculosis, Meningeal/	7606
2	((Tubercul* or TB) adj4 (meningitis or meningeal or meninges or meningitic or pachymening* or meningoencepha*)).tw,kf.	8600
3	Tuberculosis, Central Nervous System/	488
4	tuberculoma, intracranial/	695
5	tuberculoma/ and (intracranial* or cranial* or brain or midbrain or spinal cord* or cereb* or cns or central nervous or nervous system or pituitary or radiculomyelitis or arachnoiditis or myeloradiculopathy or neuro* or nerve*).tw,kf.	776
6	((intracranial* or cranial* or brain or midbrain or spinal cord* or cereb* or cns or central nervous or nervous system or pituitary or radiculomyelitis or arachnoiditis or myeloradiculopathy or neuro* or nerve*) adj6 (tubercul* or TB)).tw,kf.	6243
7	(Arachnoiditis/ or Meningoencephalitis/) and (tubercul* or TB).mp.	521
8	TBM.tw,kf.	2712
9	Neurotuberculosis.tw,kf.	167
10	or/1-9	15690
11	(HRZE* or HRZS* or RHZE*).tw,kf.	134
12	(4-FDC* or 4FDC* or 4DC* or four drug*).tw,kf.	4909
13	ethambutol/ or isoniazid/ or pyrazinamide/ or rifampin/ or exp Streptomycin/	57066
14	(ethambutol or myambutol or isoniazid or Isonicotinic acid or pyrazinamide or pyrazinoic acid or rifampicin or rifampin or streptomycin).tw,kf.	70990
15	Bedaquiline.tw,kf.	1514
16	Linezolid/	3939
17	(Linezolid or LZD or Zyvox).tw,kf.	8670
18	exp Fluoroquinolones/	39652
19	(Fluoroquinolone* or Ciprofloxacin or Fleroxacin or Enoxacin or Enrofloxacin or Gatifloxacin or Gemifloxacin or Moxifloxacin or Norfloxacin or Ofloxacin or Levofloxacin or Pefloxacin).tw,kf.	71432
20	Clofazimine/	1422
21	(Clofazimine or Lamprene).tw,kf.	1863
22	Cycloserine/	2570
23	(Cycloserine or Seromycin).tw,kf.	3061
24	terizidone.tw,kf.	66
25	(Delamanid or Deltyba).tw,kf.	546
26	exp Imipenem/	4749

27	(Imipenem or Imipemide).tw,kf.	13535
28	Meropenem/	4060
29	(meropenem or Merrem).tw,kf.	11427
30	Amikacin/	4748
31	Amikacin.tw,kf.	12351
32	Prothionamide/	199
33	(Prothionamide or Protionamide).tw,kf.	294
34	Ethionamide/	1343
35	Ethionamide.tw,kf.	1110
36	Aminosalicylic Acid/	3939
37	(Paser or Aminosalicylic or paraAminosalicylic).mp. or pas.ti.	11546
38	Pretomanid.tw,kf.	344
39	or/11-38	202537
40	10 and 39	2335
41	randomized controlled trial.pt.	633327
42	controlled clinical trial.pt.	95672
43	randomized.ab.	682739
44	placebo.ab.	256618
45	drug therapy.fs.	2789260
46	randomly.ab.	454869
47	trial.ab.	741724
48	groups.ab.	2819560
49	41 or 42 or 43 or 44 or 45 or 46 or 47 or 48	6226769
50	40 and 49 [limited to RCTS using the Cochrane Highly Sensitive Search Strategy for identifying randomized trials in MEDLINE_ sensitivity-maximizing version _2023 revision]	767

Adjunctive therapy

1	Tuberculosis, Meningeal/	7606
2	((Tubercul* or TB) adj4 (meningitis or meningeal or meninges or meningitic or pachymening* or meningoencepha*)).tw,kf.	8600
3	Tuberculosis, Central Nervous System/	488
4	tuberculoma, intracranial/	695
5	tuberculoma/ and (intracranial* or cranial* or brain or midbrain or spinal cord* or cereb* or cns or central nervous or nervous system or pituitary or radiculomyelitis or arachnoiditis or myeloradiculopathy or neuro* or nerve*).tw,kf.	776
6	((intracranial* or cranial* or brain or midbrain or spinal cord* or cereb* or cns or central nervous or nervous system or pituitary or radiculomyelitis or arachnoiditis or myeloradiculopathy or neuro* or nerve*) adj6 (tubercul* or TB)).tw,kf.	6243
7	(Arachnoiditis/ or Meningoencephalitis/) and (tubercul* or TB).mp.	521
8	TBM.tw,kf.	2712
9	Neurotuberculosis.tw,kf.	167
10	or/1-9	15690
11	exp Adrenal Cortex Hormones/	435472
12	exp Steroids/	948912
13	(Corticosteroid* or steroid* or glucocorticoid* or adrenal cortical hormone* or adrenocortical hormone* or adrenocorticosteroid* or corticoid*).tw,kf.	470600
14	(prednisolone or methylprednisolone or prednisone or dexamethasone or hydrocortisone).tw,kf.	166740
15	11 or 12 or 13 or 14 [all corticosteroid terms question 1]	1323680
16	Thalidomide/	10100
17	(sedoval* or thalidomid* or thalomid*).tw,kf.	9230
18	exp Aspirin/	49896
19	(acetylsalicylic acid or acetysal* or acylpyrin* or aloxiprimum* or aspirin* or colfarit* or dispril* or easprin* or ecotrin* or endosprin* or magnecyl* or micristin* or polopirin* or polopiryna* or solprin* or solupsan* or zorprin*).tw,kf.	68176
20	exp Infliximab/	12641

21	(inflectra* or infliximab* or remicade* or renflexis*).tw,kf.	16109
22	exp Cyclophosphamide/	58798
23	(cyclophosphamide* or cyclophosphane* or cytophosphan* or cytoxan* or endoxan* or neosar* or procytox* or sendoxan*).tw,kf.	59013
24	Interleukin 1 Receptor Antagonist Protein/	6186
25	(anakinra* or antril* or kineret* or recombinant interleukin 1 receptor antagonist*).tw,kf.	3093
26	or/16-25 [other adjuvant therapies qu 2]	204755
27	Antiretroviral Therapy, Highly Active/	22846
28	anti-retroviral agents/	13739
29	exp anti-hiv agents/ [includes individual drugs]	79692
30	anti-retroviral*.ti,ab,kf,rn.	17931
31	antiretroviral*.ti,ab,kf,rn.	78638
32	HAART.ti,ab,kf,rn.	12886
33	(ART not "state of the art").ti,ab,kf,rn.	68544
34	(ARV or ARVs).ti,ab,kf,rn.	4865
35	(zidovudine or lamivudine or stavudine or didanosine or emtricitabine or nevirapine or efavirenz or tenofovir or abacavir or atazanavir or lopinavir or ritonavir or darunavir or fosamprenavir or indinavir or saquinavir or nelfinavir or tipranavir or trizivir or combivir or kaletra or truvada or duovir or viraday or triomune or odivir or raltegravir or dolutegravir or maraviroc).ti,ab,kf,rn.	52438
36	or/27-35 [Question 3 all ART results. ART search terms taken from Burke et al. 2021 doi: 10.1002/jia2.25772.]	190799
37	Immune Reconstitution Inflammatory Syndrome/	1311
38	iris.tw,kf.	24996
39	(Immune adj2 (recovery or reconstitution or restoration or rebound) adj3 (disease* or syndrome* or illness or reaction*)).tw,kf.	2671
40	((Steroid withdrawal or Immunorestitution) adj2 (disease or syndrome)).tw,kf.	64
41	Immune response reaction*.tw,kf.	24
42	(paradoxical adj4 (inflammat* or worsening or reaction* or manifestation* or response or deterioration)).tw,kf.	2602
43	or/37-42 [question 4 - All Iris results]	29096
44	10 and 15 [Question 1 - TBM and steroids]	1289
45	10 and 26 [Question 2 - TBM and other adjunctive therapies]	155
46	10 and 36 [Question 3 - TBM and ART]	309
47	10 and 43 [Question 4 TBM and IRIS]	271

Neurocritical and neurosurgical care

1	Tuberculosis, Meningeal/	7606
2	((Tubercul* or TB) adj4 (meningitis or meningeal or meninges or meningitic or pachymening* or meningoencepha*)).tw,kf.	8600
3	Tuberculosis, Central Nervous System/	488
4	tuberculoma, intracranial/	695
5	tuberculoma/ and (intracranial* or cranial* or brain or midbrain or spinal cord* or cereb* or cns or central nervous or nervous system or pituitary or radiculomyelitis or arachnoiditis or myeloradiculopathy or neuro* or nerve*).tw,kf,hw.	906
6	((intracranial* or cranial* or brain or midbrain or spinal cord* or cereb* or cns or central nervous or nervous system or pituitary or radiculomyelitis or arachnoiditis or myeloradiculopathy or neuro* or nerve*) adj6 (tubercul* or TB)).tw,kf,hw.	7130
7	(Arachnoiditis/ or Meningoencephalitis/) and (tubercul* or TB).mp.	521
8	TBM.tw,kf.	2712
9	Neurotuberculosis.tw,kf.	167
10	or/1-9 [all searches on tbm combined]	16202
11	exp Hydrocephalus/	27105
12	hydrocephal*.tw,kf.	33732
13	Intracranial Hypertension/	6202

14	((elevat* or increase* or raised or high) adj3 (intracranial pressure or cranial pressure or icp)).tw,kf.	14376
15	((intracranial or endocranial) adj2 hypertension).tw,kf.	10597
16	(cranial adj2 hypertension).tw,kf.	129
17	or/11-16 [all searches on hydrocephalus]	62976
18	10 and 17 [tbm and hydrocephalus]	960
19	Spinal Puncture/	6982
20	(Lumbar puncture* or spinal tap or spinal taps or spinal puncture*).tw,kf.	12073
21	Furosemide/	12501
22	(Furosemid* or Frusemid* or lasix*).tw,kf.	14982
23	Acetazolamide/	7176
24	(Acetazolamid* or Diamox* or Diacarb*).tw,kf.	8555
25	cerebrospinal fluid shunts/ or ventriculoperitoneal shunt/ or ventriculostomy/	14565
26	su.fs.	2375218
27	(surg* or neurosurg*).tw,kf.	2638106
28	(drain* or ventriculostom*).tw,kf.	171142
29	shunt*.tw,kf.	76808
30	((Cerebrospinal fluid or CSF) adj2 diversion*).tw,kf.	1652
31	or/19-30 [medical and surgical management terms]	3988788
32	18 and 31 [Qu 1 and 2 tbm and hydrocephalus and medical/surgical management]	443
33	tuberculoma, intracranial/	695
34	(tuberculoma/ or tuberculoma*.tw,kf.) and (intracranial* or cranial* or brain or midbrain or spinal cord* or cereb* or cns or central nervous or nervous system or pituitary or radiculomyelitis or arachnoiditis or myeloradiculopathy or neuro* or nerve* or Intradural or extramedullary or intramedullary or medulla* or epidural*).tw,kf,hw.	2220
35	(((tuberculous or tb) adj3 granuloma*) and (intracranial* or cranial* or brain or midbrain or spinal cord* or cereb* or cns or central nervous or nervous system or pituitary or radiculomyelitis or arachnoiditis or myeloradiculopathy or neuro* or nerve* or Intradural or extramedullary or intramedullary or medulla* or epidural*)).tw,kf,hw.	98
36	33 or 34 or 35 [tuberculoma terms]	2437
37	su.fs.	2375218
38	(surg* or neurosurg*).tw,kf.	2638106
39	Resection*.mp. [mp=title, book title, abstract, original title, name of substance word, subject heading word, floating sub-heading word, keyword heading word, organism supplementary concept word, protocol supplementary concept word, rare disease supplementary concept word, unique identifier, synonyms, population supplementary concept word, anatomy supplementary concept word]	395840
40	(debulk* or remov*).tw,kf.	825177
41	excision.tw,kf.	148048
42	biops*.tw,kf.	502590
43	or/37-42	4905030
44	36 and 43 [question 3]	837
45	((tubercul* or tb or tbm) adj6 abscess*).mp.	2703
46	(exp Tuberculosis/ or Mycobacterium tuberculosis/) and (abscess/ or brain abscess/ or epidural abscess/)	1854
47	45 or 46	3675
48	(intracranial* or cranial* or brain or midbrain or spinal* or paraspinal* or cereb* or cns or central nervous or pituitary or radiculomyelitis or arachnoiditis or cerebell* or neuro* or epidural or nervous system or myeloradiculopathy or nerve* or Intradural or extramedullary or intramedullary or medulla* or subarachnoid*).mp.	5018907
49	47 and 48	1294
50	((tubercul* or tbm or tb) adj1 (intracranial* or cranial* or brain or midbrain or spinal* or paraspinal* or cereb* or cns or central nervous or pituitary or radiculomyelitis or arachnoiditis or cerebell* or neuro* or epidural or nervous system or myeloradiculopathy or nerve* or Intradural or extramedullary or intramedullary or medulla* or subarachnoid*)).mp. [mp=title, book title, abstract, original title, name of substance word, subject heading word, floating sub-heading word, keyword heading word, organism supplementary concept word, protocol supplementary concept word,	7780

	rare disease supplementary concept word, unique identifier, synonyms, population supplementary concept word, anatomy supplementary concept word]	
51	abscess*.mp. [mp=title, book title, abstract, original title, name of substance word, subject heading word, floating sub-heading word, keyword heading word, organism supplementary concept word, protocol supplementary concept word, rare disease supplementary concept word, unique identifier, synonyms, population supplementary concept word, anatomy supplementary concept word]	110087
52	50 and 51	1330
53	49 or 52	1722
54	su.fs.	2375218
55	(surg* or neurosurg*).tw,kf.	2638106
56	Resection*.tw,kf.	392396
57	exp Drainage/	67698
58	drain*.tw,kf.	168372
59	incision.tw,kf.	73605
60	debridement.tw,kf.	34706
61	debulk*.tw,kf.	9958
62	biops*.tw,kf.	502590
63	puncture*.tw,kf.	60262
64	evacuat*.tw,kf.	26782
65	fine needle aspiration.tw,kf.	33904
66	or/54-65	4444735
67	53 and 66 [Question 4]	934
68	Hyponatremia/	10579
69	(Hyponatraemi* or hyponatriaemi* or hyponatriemia* or hyponatremi* or hyposodium?emi*).tw,kf.	15739
70	(low adj3 sodium).tw,kf.	6903
71	((derange* or abnormal*) adj2 sodium).mp. [mp=title, book title, abstract, original title, name of substance word, subject heading word, floating sub-heading word, keyword heading word, organism supplementary concept word, protocol supplementary concept word, rare disease supplementary concept word, unique identifier, synonyms, population supplementary concept word, anatomy supplementary concept word]	585
72	(sodium concentration or sodium level*).tw,kf.	9663
73	cerebral salt wasting.tw,kf.	427
74	Inappropriate ADH Syndrome/	2932
75	siadh.tw,kf.	2116
76	(inappropriate adj2 (antidiure* or anti-diure* or ADH or vasopressin secretion)).tw,kf.	2472
77	siad.tw,kf.	309
78	or/68-77 [all hyponatraemia terms]	33347
79	10 and 78 [question 5]	149
80	Seizures/	66489
81	seizure*.tw,kf.	156253
82	convuls*.tw,kf.	33216
83	exp Epilepsy/	132414
84	(epileptic* or epilepsy).tw,kf.	171063
85	or/80-84 [all seizure terms]	292908
86	10 and 85 [question 6]	651

Supplementary data 2

For the following questions, no eligible studies were returned, or the studies identified did not answer the PICO question:

Diagnostics PICO 1: How accurate are CSF microscopy and biochemistry for the diagnosis of TBM?

Diagnostics PICO 4: How accurate is neuroimaging for the diagnosis of TBM?

Anti-TB chemotherapy PICO 4: Is treatment duration less than 12 months effective in TBM?

Neurocritical and neurosurgical care PICO 1: Should active management (medical and/or surgical) or standard of care be used in individuals with TB meningitis and hydrocephalus/raised ICP?

Neurocritical and neurosurgical care PICO 3: Should surgical management of tuberculomas with or without TBM occur at time of diagnosis or after medical treatment failure?

Neurocritical and neurosurgical care PICO 4: Should surgical management of TB abscesses with or without TBM occur at time of diagnosis or after medical treatment failure?

Neurocritical and neurosurgical care PICO 5: Should the management of hyponatremia in patients with TBM be based on aetiology?

Neurocritical and neurosurgical care PICO 6: Should all patients with TB meningitis be assessed for clinical and sub-clinical seizures?

Supplementary data 3

Literature searches were performed using Medline (via Ovid), Embase, Cochrane CENTRAL, and WHO Global Index Medicus, on 2nd December 2021, followed by an updated search on 24th July 2023.

A final search performed on March 11th 2025 (from July 2023 to March 11th 2025) identified 449 abstracts. These new abstracts were screened by a single researcher. After abstract screening for relevance to PICO questions, fifteen full texts were reviewed. This review, and that of other manuscripts identified from this time period, resulted in the inclusion of two additional manuscripts.

1. Kinetic Determination of Cerebrospinal Fluid Adenosine Deaminase Activity for the Diagnosis of Tuberculous Meningitis.[71]
V. Chotmongkol et al.
American Journal of Tropical Medicine & Hygiene 2024 Vol. 111 Issue 5 Pages 1024-1026.

2. Safety and Tolerability of a Short Course of Linezolid for the Treatment of Predominantly Moderate to Severe Tuberculous Meningitis in Adults with HIV.[83]
FC Chow et al.
The Journal of infectious diseases. 2025 Feb 17:jiaf089.

Supplementary data 4

Additional adjunctive therapies that were evaluated were as follows:

Thalidomide

We evaluated whether thalidomide, a tumour necrosis factor (TNF)-α inhibitor, should be initiated adjunctive to tuberculosis treatment for paradoxical CNS tuberculosis to reduce the mortality and morbidity caused by an overabundance of TNF-α. A randomised trial (n=47) was terminated early using high dose thalidomide (24mg/kg/day) due to adverse effects and mortality in the thalidomide arm.[120] Subsequently, in the same setting observational studies (n=42) demonstrated low dosage adjunctive thalidomide (2-5 mg/kg/day) to be safe and effective in treating tuberculosis mass lesions and blindness related to optochiasmatic arachnoiditis, with good clinic-radiological correlation.[121,122] Available data are mostly in the paediatric age group, and adequately powered, randomised controlled trials in both adults and children are required in multiple geographic settings to confirm efficacy and safety.

Infliximab

We evaluated whether infliximab, an anti-TNF-α monoclonal antibody, should be initiated adjunctive to tuberculosis treatment for paradoxical central nervous system tuberculosis to reduce the mortality and morbidity caused by an overabundance of TNF-α. An observational case series (n=9) showed that infliximab (3 doses of 5-10mg/kg)was safe and effective in treating tuberculosis mass lesions and blindness related to opto-chiasmatic arachnoiditis.[123,124] In a matched retrospective cohort study (n=30), adjunctive infliximab (10mg/kg 1-3 doses, 4 weeks apart) was proposed to be effective and safe in adults with severe, complicated central nervous system tuberculosis despite optimal anti-TB treatment and corticosteroids.[125] Adequately powered, randomised controlled trials are required in both adults and children with central nervous system tuberculosis, with and without HIV co-infection, in multiple geographic settings, to confirm efficacy findings and demonstrate safety.

Cyclophosphamide

We evaluated whether cyclophosphamide should be initiated adjunctive to tuberculosis treatment for paradoxical central nervous system tuberculosis (symptomatic immune-mediated vasculitis and spinal tuberculosis). Two case reports demonstrated vascular flow improvement and resolution of ischaemia after cyclophosphamide initiation in adults with symptomatic, immune-mediated vasculitis secondary to tuberculous meningitis,[126,127] and also in a case series of four patients with refractory spinal tuberculosis, all of whom had received high-dose corticosteroids. Although clinico-radiological improvement on cyclophosphamide was demonstrated,[128] caution is advised due to the risk of reactivation of tuberculosis. Adequately powered, randomised controlled trials are required in both adults and children with central nervous system tuberculosis, with and without HIV co-infection, in multiple geographic settings, to confirm efficacy findings and demonstrate safety.

Anakinra

We evaluated whether anakinra should be initiated as an adjunct to tuberculosis treatment for paradoxical central nervous system tuberculosis to reduce the mortality and morbidity of interleukin-1 mediated inflammation. A report of two cases of central nervous system tuberculosis and HIV co-infection, with worsening paradoxical inflammation despite protracted high-dose corticosteroids, demonstrated control of paradoxical inflammation and symptomatic improvement after anakinra initiation.[129] In another case series of adults with paradoxical TB, without HIV co-infection, of which two cases were of tuberculous meningitis, anakinra demonstrated clinic-radiological improvement in one, while in the other case anakinra was stopped on day three due to neutropaenia.[130] Adequately powered, randomised controlled trials are required in both adults and children with central nervous system tuberculosis, with and without HIV co-infection, in multiple geographic settings, to confirm efficacy findings and demonstrate safety.

Interferon-gamma

We evaluated whether interferon gamma should be initiated as an adjunct to tuberculosis treatment for paradoxical central nervous system tuberculosis to reduce the mortality and morbidity by modulating the host inflammatory response. A report of two adults with multiple tuberculomas of the brainstem and tuberculous abscesses respectively, despite adequate anti-tuberculosis treatment, demonstrated near complete resolution without serious side effects after subcutaneous interferon gamma initiation.[131] Adequately powered, randomised controlled trials are required in both adults and children with central nervous system tuberculosis, with and without HIV co-infection, in multiple geographic settings, to confirm efficacy findings and demonstrate safety.

References

1 Chen Y, Wang Y, Liu X, et al. Comparative diagnostic utility of metagenomic next-generation sequencing, GeneXpert, modified Ziehl-Neelsen staining, and culture using cerebrospinal fluid for tuberculous meningitis: A multi-center, retrospective study in China. *J Clin Lab Anal* 2022; 36.

2 de Almeida SM, Kussen GMB, Cogo L, Carvalho JH, Nogueira K. Diagnostic characteristics of Xpert MTB/RIF assay for the diagnosis of tuberculous meningitis and rifampicin resistance in Southern Brazil. *Arq Neuropsiquiatr* 2020; 78: 700–7.

3 Donovan J, Thu DDA, Phu NH, et al. Xpert MTB/RIF Ultra versus Xpert MTB/RIF for the diagnosis of tuberculous meningitis: a prospective, randomised, diagnostic accuracy study. *Lancet Infect Dis* 2020; 20: 299–307.

4 Li X, Du W, Wang Y, et al. Rapid Diagnosis of Tuberculosis Meningitis by Detecting *Mycobacterium tuberculosis* Cell-Free DNA in Cerebrospinal Fluid. *Am J Clin Pathol* 2020; 153: 126–30.

5 Wang S, Chen Y, Wang D, et al. The Feasibility of Metagenomic Next-Generation Sequencing to Identify Pathogens Causing Tuberculous Meningitis in Cerebrospinal Fluid. *Front Microbiol* 2019; 10.

6 A J, Ratageri VH, Illalu S, Fattepur SR, Wari PK. The Utility of CSF Xpert MTB/RIF in Diagnosis of Tubercular Meningitis in Children. *Indian J Pediatr* 2019; 86: 1089–93.

7 Metcalf T, Soria J, Montano SM, et al. Evaluation of the GeneXpert MTB/RIF in patients with presumptive tuberculous meningitis. *PLoS One* 2018; 13.

8 Cresswell F V., Bangdiwala AS, Bahr NC, et al. Tuberculous meningitis diagnosis and outcomes during the xpert MTB/Rif era: A 6.5-year cohort study in Uganda. *Wellcome Open Res* 2018; 3.

9 Heemskerk AD, Donovan J, Thu DDA, et al. Improving the microbiological diagnosis of tuberculous meningitis: A prospective, international, multicentre comparison of conventional and modified Ziehl–Neelsen stain, GeneXpert, and culture of cerebrospinal fluid. *J Infect* 2018; 77: 509–515.

10 Wang T, Feng G-D, Pang Y, et al. Sub-optimal Specificity of Modified Ziehl-Neelsen Staining for Quick Identification of Tuberculous Meningitis. *Front Microbiol* 2016; 7: 2096.

11 Feng G, Shi M, Ma L, et al. Diagnostic accuracy of intracellular *mycobacterium tuberculosis* detection for tuberculous meningitis. *Am J Respir Crit Care Med* 2014; 189: 475–81.

12 Patel VB, Theron G, Lenders L, et al. Diagnostic accuracy of quantitative PCR (Xpert MTB/RIF) for tuberculous meningitis in a high burden setting: a prospective study. *PLoS Med* 2013; 10.

13 Sehgal V, Sharma M, LNU P, et al. Comparison of Protein B Polymerase Chain Reaction (PCR) With IS6110 PCR for Diagnosis of Tuberculous Meningitis Patients. *Cureus* 2023; 15.

14 Hai HT, Sabiiti W, Thu DDA, et al. Evaluation of the molecular bacterial load assay for detecting viable *Mycobacterium tuberculosis* in cerebrospinal fluid before and during tuberculous meningitis treatment. *Tuberculosis (Edinb)* 2021; 128.

15 Agarwal S, Saini AG, Dhawan S, Khadwal A, Sharma K, Singhi P. Comparative evaluation of IS6110 and protein antigen b PCR in cerebrospinal fluid for rapid diagnosis of tuberculous meningitis in children. *J Med Microbiol* 2020; 69: 979–85.

16 Chaidir L, Annisa J, Dian S, et al. Microbiological diagnosis of adult tuberculous meningitis in a ten-year cohort in Indonesia. *Diagn Microbiol Infect Dis* 2018; 91: 42–6.

17 Rufai SB, Singh A, Singh J, et al. Diagnostic usefulness of Xpert MTB/RIF assay for detection of tuberculous meningitis using cerebrospinal fluid. *J Infect* 2017; 75: 125–31.

18 Solomons RS, Visser DH, Marais BJ, Schoeman JF, Van Furth AM. Diagnostic accuracy of a uniform research case definition for TBM in children: a prospective study. *Int J Tuberc Lung Dis* 2016; 20: 903–8.

19 Bahr NC, Tugume L, Rajasingham R, et al. Improved diagnostic sensitivity for tuberculous meningitis with Xpert® MTB/RIF of centrifuged CSF. *Int J Tuberc Lung Dis* 2015; 19: 1209–15.

20 Erdem H, Ozturk-Engin D, Elaldi N, et al. The microbiological diagnosis of tuberculous meningitis: results of Haydarpasa-1 study. *Clin Microbiol Infect* 2014; 20: O600–8.

21 Kusum S, Aman S, Pallab R, et al. Multiplex PCR for rapid diagnosis of tuberculous meningitis. *J Neurol* 2011; 258: 1781–7.

22 Caws M, Ha DTM, Torok E, et al. Evaluation of the MODS culture technique for the diagnosis of tuberculous meningitis. *PLoS One* 2007; 2: 1173.

23 Chua H, Tay L, Wang S, Chan Y. Use of ligase chain reaction in early diagnosis of tuberculous meningitis. *Ann Acad Med Singap* 2005; 34: 149–53.

24 Thwaites GE, Caws M, Chau TTH, et al. Comparison of conventional bacteriology with nucleic acid amplification (amplified mycobacterium direct test) for diagnosis of tuberculous meningitis before and after inception of antituberculosis chemotherapy. *J Clin Microbiol* 2004; 42: 996–1002.

25 Johansen IS, Lundgren B, Tabak F, et al. Improved sensitivity of nucleic acid amplification for rapid diagnosis of tuberculous meningitis. *J Clin Microbiol* 2004; 42: 3036–40.

26 Rafi A, Naghily B. Efficiency of polymerase chain reaction for the diagnosis of tuberculous meningitis. *Southeast Asian J Trop Med Public Health* 2003; 34: 357–60.

27 Bonington A, Strang J, Klapper P, et al. TB PCR in the early diagnosis of tuberculous meningitis: evaluation of the Roche semi-automated COBAS Amplicor MTB test with reference to the manual Amplicor MTB PCR test. *Tuber Lung Dis* 2000; 80: 191–6.

28 Nguyen LN, Kox LFF, Pham LD, Kuijper S, Kolk AHJ. The potential contribution of the polymerase chain reaction to the diagnosis of tuberculous meningitis. *Arch Neurol* 1996; 53: 771–6.

29 Kox L, Kuijper S, Kolk A. Early diagnosis of tuberculous meningitis by polymerase chain reaction. *Neurology* 1995; 45: 2228–32.

30 Krishnakumariamma K, Ellappan K, Muthuraj M, Tamilarasu K, Kumar SV, Joseph NM. Diagnostic performance of real time PCR for the detection of *Mycobacterium tuberculosis* in cerebrospinal fluid samples. *Indian J Med Microbiol* 2023; 42: 7–11.

31 Rai A, Prasad R, Das BK, Anupurba S, Singh UK. Cerebrospinal fluid Gene XPERT (CBNAAT) in children with tuberculous meningitis. *J Clin Tuberc Other Mycobact* Dis 2021; 24.

32 Yu G, Wang X, Zhu P, Shen Y, Zhao W, Zhou L. Comparison of the efficacy of metagenomic next-generation sequencing and Xpert MTB/RIF in the diagnosis of tuberculous meningitis. *J Microbiol Methods* 2021; 180.

33 Sharma K, Sharma M, Modi M, et al. Comparative analysis of Truenat MTB Plus and GeneXpert Ultra in diagnosing tuberculous meningitis. *Int J Tuberc Lung Dis* 2021; 25: 626-631.

34 Huang M, Wang G, Sun Q, et al. Diagnostic accuracy of Xpert MTB/RIF Ultra for tuberculous meningitis in a clinical practice setting of China. *Diagn Microbiol Infect Dis* 2021; 100: 115306.

35 Ajbani K, Kazi M, Agrawal U, et al. Evaluation of CSF pyrosequencing to diagnose tuberculous meningitis: A retrospective diagnostic accuracy study. *Tuberculosis (Edinb)* 2021; 126: 102048.

36 Sharma K, Sharma M, Shree R, et al. Xpert MTB/RIF ultra for the diagnosis of tuberculous meningitis: A diagnostic accuracy study from India. *Tuberculosis (Edinb)* 2020; 125: 101990.

37 Cresswell F V, Tugume L, Bahr NC, et al. Xpert MTB/RIF Ultra for the diagnosis of HIV-associated tuberculous meningitis: a prospective validation study. *Lancet Infect Dis* 2020; 20: 308–317.

38 Azevedo RG, Dinallo FS, De Laurentis LS, Boulware DR, Vidal JE. Xpert MTB/RIF® assay for the diagnosis of HIV-related tuberculous meningitis in São Paulo, Brazil. *Int J Tuberc Lung Dis* 2018; 22: 706–7.

39 Sharma K, Sharma M, Chaudhary L, et al. Comparative evaluation of Xpert MTB/RIF assay with multiplex polymerase chain reaction for the diagnosis of tuberculous meningitis. *Tuberculosis (Edinb)* 2018; 113: 38–42.

40 Bahr NC, Nuwagira E, Evans EE, et al. Diagnostic accuracy of Xpert MTB/RIF Ultra for tuberculous meningitis in HIV-infected adults: a prospective cohort study. *Lancet Infect Dis* 2018; 18: 68–75.

41 Solomons RS, Visser DH, Friedrich SO, et al. Improved diagnosis of childhood tuberculous meningitis using more than one nucleic acid amplification test. *Int J Tuberc Lung Dis* 2015; 19: 74–80.

42 Patel VB, Connolly C, Singh R, et al. Comparison of amplicor and GeneXpert MTB/RIF tests for diagnosis of tuberculous meningitis. *J Clin Microbiol* 2014; 52: 3777–80.

43 Pradhan NN, Paradkar MS, Kagal A, et al. Performance of Xpert® MTB/RIF and Xpert® Ultra for the diagnosis of tuberculous meningitis in children. *Int J Tuberc Lung Dis* 2022; 26: 317–25.

44 Wakode P, Siddaiah N, Manjunath N, Bahubali VKH. GeneXpert: A Rapid and Supplementary Diagnostic Tool for Tuberculous Meningitis, Experience from Tertiary Neurocenter. *J Neurosci Rural Pract* 2022; 13: 204–10.

45 Krishnakumariamma K, Ellappan K, Muthuraj M, Tamilarasu K, Kumar SV, Joseph NM. Molecular diagnosis, genetic diversity and drug sensitivity patterns of Mycobacterium tuberculosis strains isolated from tuberculous meningitis patients at a tertiary care hospital in South India. PLoS One 2020; 15: e0240257.

46 Singh Rathour J, Mantan M, Khanna A, Hanif M. Evaluation of GeneXpert Assay in Extrapulmonary Tuberculosis in Children. *J Evolution Med Dent Sci* 2019; 8.

47 Pink F, Brown TJ, Kranzer K, Drobniewski F. Evaluation of Xpert MTB/RIF for Detection of *Mycobacterium tuberculosis* in Cerebrospinal Fluid. *J Clin Microbiol* 2016; 54: 809–11.

48 Yadav B, Sharma M, Singla N, et al. Molecular diagnosis of Tuberculous meningitis: sdaA-based multi-targeted LAMP and GeneXpert Ultra. *Tuberculosis (Edinb)* 2023; 140: 102339.

49 Quinn CM, Kagimu E, Okirworth M, et al. Fujifilm SILVAMP TB LAM Assay on Cerebrospinal Fluid for the Detection of Tuberculous Meningitis in Adults With Human Immunodeficiency Virus. *Clin Infect Dis* 2021; 73: E3428–34.

50 Shao L, Qiu C, Zheng L, et al. Comparison of diagnostic accuracy of the GeneXpert Ultra and cell-free nucleic acid assay for tuberculous meningitis: A multicentre prospective study. *Int J Infect Dis* 2020; 98: 441–6.

51 Chin JH, Musubire AK, Morgan N, et al. Xpert MTB/RIF Ultra for Detection of Mycobacterium tuberculosis in Cerebrospinal Fluid. *J Clin Microbiol* 2019; 5: e00249-19

52 Wang G, Wang S, Jiang G, et al. Xpert MTB/RIF Ultra improved the diagnosis of paucibacillary tuberculosis: A prospective cohort study. J Infect 2019; 78: 311-316.

53 Kwizera R, Cresswell F V, Mugumya G, et al. Performance of Lipoarabinomannan Assay using Cerebrospinal fluid for the diagnosis of Tuberculous meningitis among HIV patients. *Wellcome Open Res* 2019; 4: 123.

54 Siddiqi OK, Birbeck GL, Ghebremichael M, et al. Prospective Cohort Study on Performance of Cerebrospinal Fluid (CSF) Xpert MTB/RIF, CSF Lipoarabinomannan (LAM) Lateral Flow Assay (LFA), and Urine LAM LFA for Diagnosis of Tuberculous Meningitis in Zambia. *J Clin Microbiol* 2019; 57: e00652-19

55 Chander A, Shrestha CD. Cerebrospinal fluid adenosine deaminase levels as a diagnostic marker in tuberculous meningitis in adult Nepalese patients. *Asian Pac J Trop Dis* 2013; 3: 16–19.

56 Chotmongkol V, Teerajetgul Y, Yodwut C. Cerebrospinal fluid adenosine deaminase activity for the diagnosis of tuberculous meningitis in adults. *Southeast Asian J Trop Med Public Health* 2006; 37: 948–52.

57 Coovadia YM, Dawood A, Ellis ME, Coovadia HM, Daniel TM. Evaluation of adenosine deaminase activity and antibody to *Mycobacterium tuberculosis* antigen 5 in cerebrospinal fluid and the radioactive bromide partition test for the early diagnosis of tuberculosis meningitis. *Arch Dis Child* 1986; 61: 428–35.

58 Donald P, Malan C, van der Walt A, Schoeman J. The simultaneous determination of cerebrospinal fluid and plasma adenosine deaminase activity as a diagnostic aid in tuberculous meningitis. *S Afr Med J* 1986; 69: 505–7.

59 Eintracht S, Silber E, Sonnenberg P, Koornhof HJ, Saffer D. Analysis of adenosine deaminase isoenzyme-2 (ADA(2)) in cerebrospinal fluid in the diagnosis of tuberculosis meningitis. *J Neurol Neurosurg Psychiatry* 2000; 69: 137–8.

60 Ghosh GC, Sharma B, Gupta BB. CSF ADA Determination in Early Diagnosis of Tuberculous Meningitis in HIV-Infected Patients. *Scientifica (Cairo)* 2016; 2016: 5820823.

61 Kashyap RS, Kainthla RP, Mudaliar A V., Purohit HJ, Taori GM, Daginawala HF. Cerebrospinal fluid adenosine deaminase activity: a complimentary tool in the early diagnosis of tuberculous meningitis. *Cerebrospinal Fluid Res* 2006; 3: 5.

62 Kwon JS, Park JH, Kim JY, et al. Diagnostic Usefulness of Cytokine and Chemokine Levels in the Cerebrospinal Fluid of Patients with Suspected Tuberculous Meningitis. *Am J Trop Med Hyg* 2019; 101: 343.

63 López-Cortés LF, Cruz-Ruiz M, Gómez-Mateos H, et al. Adenosine deaminase activity in the CSF of patients with aseptic meningitis: utility in the diagnosis of tuberculous meningitis or neurobrucellosis. *Clin Infect Dis* 1995; 20: 525–30.

64 Mishra O, Loiwal V, Ali Z, Nath G, Chandra L. Cerebrospinal fluid adenosine deaminase activity for the diagnosis of tuberculous meningitis in children. *J Trop Pediatr* 1996; 42: 129–32.

65 Park KH, Cho OH, Lee EM, et al. T-cell-based assays on cerebrospinal fluid and PBMCs for rapid diagnosis of TB meningitis in non-HIV patients. *Eur Respir J* 2012; 39: 768–70.

66 Pettersson T, Klockars M, Weber TH, Somer H. Diagnostic value of cerebrospinal fluid adenosine deaminase determination. *Scand J Infect Dis* 1991; 23: 97–100.

67 Rana S V., Chacko F, Lal V, et al. To compare CSF adenosine deaminase levels and CSF-PCR for tuberculous meningitis. *Clin Neurol Neurosurg* 2010; 112: 424–30.

68 Ribera E, Martinez-Vazquez JM, Ocaña I, Segura RM, Pascual C. Activity of adenosine deaminase in cerebrospinal fluid for the diagnosis and follow-up of tuberculous meningitis in adults. *J Infect Dis* 1987; 155: 603–7.

69 Rohani M, Cheong Y, Rani J. The use of adenosine deaminase activity as a biochemical marker for the diagnosis of tuberculous meningitis. *Malays J Pathol* 1995; 17: 67–71.

70 Solari L, Soto A, Agapito JC, et al. The validity of cerebrospinal fluid parameters for the diagnosis of tuberculous meningitis. *Int J Infect Dis* 2013; 17: e1111-5.

71 Chotmongkol V, Thongsaen P, Sawanyawisuth K, et al. Kinetic Determination of Cerebrospinal Fluid Adenosine Deaminase Activity for the Diagnosis of Tuberculous Meningitis. *Am J Trop Med Hyg* 2024; 111: 1024-1026.

72 Desai K, Malek S, Shah N, Shah P, Joshi P, Dave J. Diagnostic evaluation of adenosine deaminase (ADA) test in the early diagnosis of tuberculous meningitis. *Int J Med Public Health* 2011; 1: 9–12.

73 Krishnaswamy R, Priyadharshini K. Diagnostic Accuracy of Cerebrospinal Fluid Adenosine Deaminase Activity in Tuberculous Meningitis. *JMSCR* 2016; 4: 12565–8.

74 Cresswell F V., Meya DB, Kagimu E, et al. High-Dose Oral and Intravenous Rifampicin for the Treatment of Tuberculous Meningitis in Predominantly Human Immunodeficiency Virus (HIV)-Positive Ugandan Adults: A Phase II Open-Label Randomized Controlled Trial. *Clin Infect Dis* 2021; 73: 876–84.

75 Heemskerk AD, Bang ND, Mai NTH, et al. Intensified Antituberculosis Therapy in Adults with Tuberculous Meningitis. *N Engl J Med* 2016; 374: 124–34.

76 Ruslami R, Ganiem AR, Dian S, et al. Intensified regimen containing rifampicin and moxifloxacin for tuberculous meningitis: an open-label, randomised controlled phase 2 trial. *Lancet Infect Dis* 2013; 13: 27–35.

77 Dian S, Yunivita V, Ganiem AR, et al. Double-Blind, Randomized, Placebo-Controlled Phase II Dose-Finding Study To Evaluate High-Dose Rifampin for Tuberculous Meningitis. *Antimicrob Agents Chemother* 2018; 62: e01014-18.

78 Davis AG, Wasserman S, Stek C, et al. A Phase 2A Trial of the Safety and Tolerability of Increased Dose Rifampicin and Adjunctive Linezolid, With or Without Aspirin, for Human Immunodeficiency Virus-Associated Tuberculous Meningitis: The LASER-TBM Trial. *Clin Infect Dis* 2023; 76: 1412–22.

79 Thwaites GE, Bhavnani SM, Chau TTH, et al. Randomized pharmacokinetic and pharmacodynamic comparison of fluoroquinolones for tuberculous meningitis. *Antimicrob Agents Chemother* 2011; 55: 3244–53.

80 Kalita J, Bhoi SK, Betai S, Misra UK. Safety and efficacy of additional levofloxacin in tuberculous meningitis: A randomized controlled pilot study. *Tuberculosis (Edinb)* 2016; 98: 1–6.

81 Kalita J, Misra UK, Prasad S, Bhoi SK. Safety and efficacy of levofloxacin versus rifampicin in tuberculous meningitis: an open-label randomized controlled trial. *J Antimicrob Chemother* 2014; 69: 2246–51.

82 Sahib A, Bhatia R, Srivastava MVP, et al. Escalate: Linezolid as an add on treatment in the intensive phase of tubercular meningitis. A randomized controlled pilot trial. *Tuberculosis (Edinb)* 2023; 142: 102351.

83 Chow FC, Kafeero P, Nakimbugwe M, et al. Safety and Tolerability of a Short Course of Linezolid for the Treatment of Predominantly Moderate to Severe Tuberculous Meningitis in Adults with HIV. *J Infect Dis* 2025: jiaf089.

84 Butov D, Feshchenko Y, Kuzhko M, et al. Effectiveness of Intravenous Isoniazid and Ethambutol Administration in Patients with Tuberculosis Meningoencephalitis and HIV Infection. *Tuberc Respir Dis (Seoul)* 2020; 83: 1–8.

85 Steeken W, Wolinsky E, Pratt PC. Streptomycin and PAS in experimental tuberculosis of guinea pigs infected intracerebrally with virulent tubercle bacilli. *Am Rev Tuberc* 1951; 64: 87–101.

86 Chen X, Arun B, Nino-Meza OJ, et al. Dynamic PET reveals compartmentalized brain and lung tissue antibiotic exposures of tuberculosis drugs. *Nat Commun* 2024; 15.

87 Hughes IE, Smith H, Kane PO. Ethionamide: its passage into the cerebrospinal fluid in man. *Lancet* 1962; 1: 616–7.

88 Kempker RR, Smith AGC, Avaliani T, et al. Cycloserine and Linezolid for Tuberculosis Meningitis: Pharmacokinetic Evidence of Potential Usefulness. *Clin Infect Dis* 2022; 75: 682–9.

89 Donald PR, Seifart HI. Cerebrospinal fluid concentrations of ethionamide in children with tuberculous meningitis. *J Pediatr* 1989; 115: 483–6.

90 Court R, Wiesner L, Stewart A, et al. Steady state pharmacokinetics of cycloserine in patients on terizidone for multidrug-resistant tuberculosis. *Int J Tuberc Lung Dis* 2018; 22: 30–3.

91 Hwang TJ, Wares DF, Jafarov A, Jakubowiak W, Nunn P, Keshavjee S. Safety of cycloserine and terizidone for the treatment of drug-resistant tuberculosis: a meta-analysis. *Int J Tuberc Lung Dis* 2013; 17: 1257–66.

92 Abdelgawad N, Wasserman S, Abdelwahab MT, et al. Linezolid Population Pharmacokinetic Model in Plasma and Cerebrospinal Fluid Among Patients With Tuberculosis Meningitis. *J Infect Dis* 2024; 229: 1200–8.

93 Baijnath S, Moodley C, Ngcobo B, et al. Clofazimine protects against *Mycobacterium tuberculosis* dissemination in the central nervous system following aerosol challenge in a murine model. *Int J Antimicrob Agents* 2018; 51: 77–81.

94 Tucker EW, Ruiz-Bedoya CA, Mota F, et al. Linezolid does not improve bactericidal activity of rifampin-containing first-line regimens in animal models of TB meningitis. *Int J Antimicrob Agents* 2024; 63.

95 Mehta K, Balazki P, van der Graaf PH, Guo T, van Hasselt JGC. Predictions of Bedaquiline Central Nervous System Exposure in Patients with Tuberculosis Meningitis Using Physiologically based Pharmacokinetic Modeling. *Clin Pharmacokinet* 2024; 63: 657–68.

96 Upton CM, Steele CI, Maartens G, Diacon AH, Wiesner L, Dooley KE. Pharmacokinetics of bedaquiline in cerebrospinal fluid (CSF) in patients with pulmonary tuberculosis (TB). *J Antimicrob Chemother* 2022; 77: 1720–4.

97 Tucker EW, Pieterse L, Zimmerman MD, et al. Delamanid Central Nervous System Pharmacokinetics in Tuberculous Meningitis in Rabbits and Humans. Antimicrob Agents Chemother 2019; 63.

98 Upton C, Aarnoutse R, Dooley K, et al. Cerebrospinal fluid penetration of delamanid, clofazimine and terizidone in pulmonary TB patients. Abstract OA12-291-16. In: 48th Union World Conference on Lung Health. *Int J Tuberc Lung Dis* 2017; 21.

99 Mota F, Ruiz-Bedoya CA, Tucker EW, et al. Dynamic 18F-Pretomanid PET imaging in animal models of TB meningitis and human studies. *Nat Commun* 2022; 13.

100 Winter H, Ginsberg A, Egizi E, et al. Effect of a high-calorie, high-fat meal on the bioavailability and pharmacokinetics of PA-824 in healthy adult subjects. *Antimicrob Agents Chemother* 2013; 57: 5516–20.

101 Ginsberg AM, Laurenzi MW, Rouse DJ, Whitney KD, Spigelman MK. Safety, tolerability, and pharmacokinetics of PA-824 in healthy subjects. *Antimicrob Agents Chemother* 2009; 53: 3720–5.

102 Baijnath S, Naiker S, Shobo A, et al. Evidence for the presence of clofazimine and its distribution in the healthy mouse brain. *J Mol Histol* 2015; 46: 439–42.

103 Irwin SM, Gruppo V, Brooks E, et al. Limited activity of clofazimine as a single drug in a mouse model of tuberculosis exhibiting caseous necrotic granulomas. *Antimicrob Agents Chemother* 2014; 58: 4026–34.

104 Swanson R V., Adamson J, Moodley C, et al. Pharmacokinetics and pharmacodynamics of clofazimine in a mouse model of tuberculosis. *Antimicrob Agents Chemother* 2015; 59: 3042–51.

105 Chotmongkol V, Jitpimolmard S, Thavornpitak Y. Corticosteroid in tuberculous meningitis. *J Med Assoc Thai* 1996; 79: 83–90.

106 Girgis NI, Farid Z, Kilpatrick ME, Sultan Y, Mikhail IA. Dexamethasone adjunctive treatment for tuberculous meningitis. *Pediatr Infect Dis J* 1991; 10: 179–83.

107 Kumarvelu S, Prasad K, Khosla A, Behari M, Ahuja GK. Randomized controlled trial of dexamethasone in tuberculous meningitis. *Tuber Lung Dis* 1994; 75: 203–7.

108 Lardizabal DV, Roxas AA Jr. Dexamethasone as adjunctive therapy in adult patients with probable tuberculous meningitis stage II and III: An open randomized controlled trial. *Philippine Journal of Neurology* 1998. https://www.herdin.ph/index.php?view=research&cid=33064 (accessed July 22, 2024).

109 Malhotra HS, Garg RK, Singh MK, Agarwal A, Verma R. Corticosteroids (dexamethasone versus intravenous methylprednisolone) in patients with tuberculous meningitis. *Ann Trop Med Parasitol* 2009; 103: 625–34.

110 Schoeman JF, Van Zyl LE, Laubscher JA, Donald PR. Effect of corticosteroids on intracranial pressure, computed tomographic findings, and clinical outcome in young children with tuberculous meningitis. *Pediatrics* 1997; 99: 226–31.

111 Thwaites GE, Bang ND, Dung NH, et al. Dexamethasone for the Treatment of Tuberculous Meningitis in Adolescents and Adults. *N Engl J Med* 2004; 351: 1741–51.

112 Donovan J, Bang ND, Imran D, et al. Adjunctive Dexamethasone for Tuberculous Meningitis in HIV-Positive Adults. *N Engl J Med* 2023; 389: 1357–67.

113 O'Toole RD, Thornton GF, Mukherjee MK, Nath RL. Dexamethasone in tuberculous meningitis. Relationship of cerebrospinal fluid effects to therapeutic efficacy. *Ann Intern Med* 1969; 70: 39–48.

114 Torok ME, Yen NTB, Chau TTH, et al. Timing of Initiation of Antiretroviral Therapy in Human Immunodeficiency Virus (HIV)-Associated Tuberculous Meningitis. *Clin Infect Dis* 2011; 52: 1374–83.

115 Misra UK, Kalita J, Nair PP. Role of aspirin in tuberculous meningitis: A randomized open label placebo controlled trial. *J Neurol Sci* 2010; 293: 12–7.

116 Mai NT, Dobbs N, Phu NH, et al. A randomised double blind placebo controlled phase 2 trial of adjunctive aspirin for tuberculous meningitis in HIV-uninfected adults. *Elife* 2018; 7: e87888.

117 Schoeman JF, Janse van Rensburg A, Laubscher JA, Springer P. The Role of Aspirin in Childhood Tuberculous Meningitis. *J Child Neurol* 2011; 26: 956–62.

118 Aranha A, Choudhary A, Bhaskar S, Gupta LN. A Randomized Study Comparing Endoscopic Third Ventriculostomy versus Ventriculoperitoneal Shunt in the Management of Hydrocephalus Due to Tuberculous Meningitis. *Asian J Neurosurg* 2018; 13: 1140–7.

119 Goyal P, Srivastava C, Ojha BK, et al. A randomized study of ventriculoperitoneal shunt versus endoscopic third ventriculostomy for the management of tubercular meningitis with hydrocephalus. *Child's Nervous System* 2014; 30: 851–7.

120 Schoeman JF, Springer P, van Rensburg AJ, et al. Adjunctive thalidomide therapy for childhood tuberculous meningitis: results of a randomized study. *J Child Neurol* 2004; 19: 250–7.

121 Schoeman JF, Fieggen G, Seller N, Mendelson M, Hartzenberg B. Intractable Intracranial Tuberculous Infection Responsive to Thalidomide: Report of Four Cases. *J Child Neurol* 2006; 21: 301–8.

122 Van Toorn R, Solomons RS, Seddon JA, Schoeman JF. Thalidomide Use for Complicated Central Nervous System Tuberculosis in Children: Insights From an Observational Cohort. *Clin Infect Dis* 2021; 72: e136–45.

123 Abo YN, Curtis N, Osowicki J, et al. Infliximab for Paradoxical Reactions in Pediatric Central Nervous System Tuberculosis. *J Pediatric Infect Dis Soc* 2021; 10: 1087–91.

124 Marais BJ, Cheong E, Fernando S, et al. Use of Infliximab to Treat Paradoxical Tuberculous Meningitis Reactions. *Open Forum Infect Dis* 2021; 8: ofaa604.

125 Manesh A, Gautam P, Kumar SSD, et al. Effectiveness of Adjunctive High-Dose Infliximab Therapy to Improve Disability-Free Survival Among Patients With Severe Central Nervous System Tuberculosis: A Matched Retrospective Cohort Study. *Clin Infect Dis* 2023; 77: 1460–7.

126 Gonzalez-Duarte A, Higuera-Calleja J, Flores F, Davila-Maldonado L, Cantú-Brito C. Cyclophosphamide treatment for unrelenting CNS vasculitis secondary to tuberculous meningitis. *Neurology* 2012; 78: 1277–8.

127 Celotti A, Vianello F, Sattin A, Malipiero G, Faggin R, Cattelan A. Cyclophosphamide immunomodulation of TB-associated cerebral vasculitis. *Infect Dis (Lond)* 2018; 50: 779–82.

128 Goyal V, Elavarasi A, Kumar A, et al. Cyclophosphamide therapy as an adjunct in refractory post-tubercular arachnoiditis. *Indian J Tuberc* 2022; 69: 325–33.

129 Keeley AJ, Parkash V, Tunbridge A, et al. Anakinra in the treatment of protracted paradoxical inflammatory reactions in HIV-associated tuberculosis in the United Kingdom: a report of two cases. *Int J STD AIDS* 2020; 31: 808–12.

130 van Arkel C, Boeree M, Magis-Escurra C, et al. Interleukin-1 receptor antagonist anakinra as treatment for paradoxical responses in HIV-negative tuberculosis patients: A case series. *Med (N Y)* 2022; 3: 603-611.e2.

131 Lee JY, Yim JJ, Yoon BW. Adjuvant interferon-γ treatment in two cases of refractory tuberculosis of the brain. *Clin Neurol Neurosurg* 2012; 114: 732–4.
